# Isolation of *Candida maltosa* strains able to achieve a high lipid productivity from malt bagasse hemicellulosic hydrolysate

**DOI:** 10.1007/s11274-026-04919-9

**Published:** 2026-03-30

**Authors:** Fernanda Pinheiro Moreira Freitas, Rodrigo Gonçalves Dias, Eduardo Luís Menezes de Almeida, Samuel Lessa Barbosa, Carina Aline Prado, Júlio César dos Santos, Silvio Silvério da Silva, Wendel Batista da Silveira

**Affiliations:** 1 Department of Microbiology, Universidade Federal de Viçosa, Viçosa, MG 36570-900 Brazil; 2 Institute of Biotechnology Applied to Agriculture (BIOAGRO), Universidade Federal de Viçosa, Viçosa, MG 36570-900 Brazil; 3 Department of Biochemistry and Molecular Biology, Universidade Federal de Viçosa, Viçosa, MG 36570-900 Brazil; 4 Department of Biotechnology, School of Engineering of Lorena – Universidade de São Paulo, Lorena, SP 12600-900 Brazil

**Keywords:** Sustainable oleochemicals, Yeast, Response surface methodology, Biorefineries

## Abstract

The rising demand for renewable and sustainable oleochemicals underscores the necessity of producing yeast oil from lignocellulosic hydrolysates. We isolated two oleaginous yeast strains of *Candida maltosa* (UFV-1 and UFV-2) able to assimilate xylose and tolerate hemicellulosic hydrolysate inhibitors (acetic acid, furfural, hydroxymethylfurfural and formic acid). To optimize growth and lipid production by *C. maltosa* in malt bagasse hemicellulosic hydrolysate, we applied a central composite rotational design with response surface methodology, evaluating initial pH and yeast extract:peptone ratio (P:YE). The maximum lipid responses were recorded in: (P:YE = 1.0:0.0 g/L, pH = 6.0) for UFV-1 and (P:YE = 0.58:0.42 g/L, pH = 6.5) for UFV-2. Biomass and lipid production were higher in the detoxified hydrolysate than in the non-detoxified hydrolysate. Biomass titers increased from 9.12 to 17.0 g/L and from 10.5 to 12.9 g/L for UFV-1 and UFV-2, respectively; whilst the lipid titers increased from 0.84 to 3.14 g/L and 0.91 to 1.58 g/L for UFV-1 and UFV-2, respectively. Importantly, the levels of 16:0, 18:0, 10:0 fatty acids increased in the detoxified medium. Cultivation of the UFV-1 strain from detoxified hydrolysate in a benchtop bioreactor improved biomass formation, achieving a lipid productivity of 0.29 ± 0.02 g/L h, highlighting its potential to produce oil from hemicellulosic hydrolysates in biorefineries.

**Graphic abstract:**

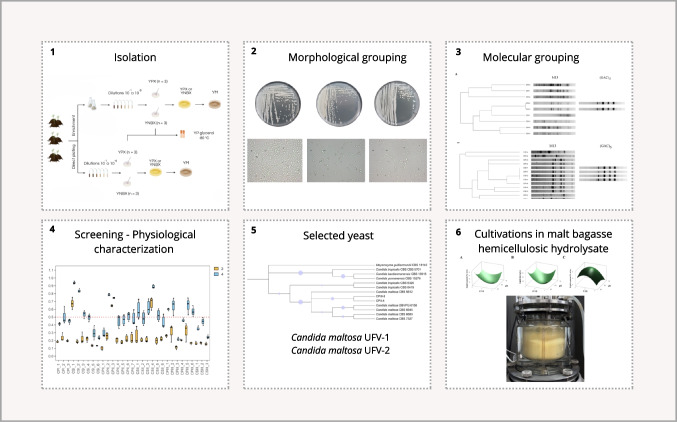

**Supplementary Information:**

The online version contains supplementary material available at 10.1007/s11274-026-04919-9.

## Introduction

Fluctuations in fossil fuel prices along with concerns about energy security and increasing awareness of the environmental impacts of CO₂ emissions, have driven the search for alternative oleochemical production processes with potential to replace petroleum-derived products. Microbial lipids, also known as single-cell oils (SCOs), stand out due to their wide applicability across various industrial sectors such as the formulation of functional foods, plant-based foods, animal feed, cosmetics, pharmaceuticals, as well as the production of fatty acid-derived biofuels and bioplastics (Costa et al. 2024; Tomás-Pejó et al. 2021). Microorganisms such as filamentous fungi, yeasts, microalgae and bacteria able to accumulate more than 20% of their dry weight (DW) as lipids are classified as oleaginous. Among them, yeasts are notable due to their high lipid yield, and ability to assimilate a wide range of carbon sources, making them attractive for biotechnological applications (Abeln and Chuck 2021; Thorpe and Ratledge 1972).

An important drawback for the industrial production of SCOs is the high cost of purified substrates such as glucose and xylose, which limits the bioprocess feasibility (Valdés et al. 2020). Low-cost lignocellulosic byproducts are alternative carbon sources for yeast cultivation in biorefineries, as they can be hydrolyzed to release fermentable sugars. Among these, malt bagasse stands out as the main solid byproduct of the brewing industry, representing approximately 85% of total solids production (Zeko-Pivac et al. 2022). It is rich in fiber, especially hemicellulose, and remains largely underutilized (Paz et al. 2019). On average, 20 kg of malt bagasse is generated per 100 L of beer produced, resulting in an estimated annual production of over 36 million tons worldwide (Zeko-Pivac et al. 2022; Nyhan et al. 2023). Furthermore, the global beer market was valued at US$839.31 billion in 2024 and is projected to reach US$1,248.3 billion by 2030, growing at a compound annual growth rate (CAGR) of 6.8% between 2025 and 2030 (Grand View Research 2025). This market expansion has increased the availability of malt bagasse, reinforcing its potential as a low-cost raw material for biotechnological applications.

Despite its high carbohydrate content, lignocellulosic biomasses are highly recalcitrant due to the complex and rigid structure of plant cell walls, which hinder microbial access to fermentable sugars. To overcome this drawback, pretreatment processes are required to disrupt the matrix and release sugars for microbial cultivation (Canilha et al. 2012; Mankar et al. 2021). Dilute acid hydrolysis is one of the most commonly used strategies, as it breaks down the structural matrix of plant cell walls, promoting partial solubilization of hemicellulose and releasing sugars such as xylose, arabinose, and glucose (Kucharska et al. 2018; Mankar et al. 2021). However, this pretreatment also generates inhibitory compounds such as furfural, hydroxymethylfurfural (HMF), and organic acids such as acetic acid and formic acid, which negatively affect microbial growth. These inhibitors interfere with enzymatic activity and microbial metabolism, reducing the efficiency of biomass conversion into biobased products (Xu et al. 2019). Among them, acetic acid is one of the most prevalent inhibitors found in hemicellulosic hydrolysates, as it is released due to hemicellulose deacetylation during pretreatment. Acetic acid, at low pH (*i.e.* < 4.76), can freely diffuse through the cell membrane in its undissociated form, causing intracellular acidification, disrupting metabolic homeostasis, and inhibiting growth (Jönsson and Martín 2016; Palmqvist and Hahn-Hägerdal 2000; Pampulha and Loureiro-Dias 2000).

Managed soils, particularly in agricultural and experimental areas, offer unique opportunities for the bioprospection of robust and productive yeast strains. The combination of management practices and the presence of lignocellulosic materials creates selective microenvironments that favor yeast communities adapted to local conditions (Niu et al. 2021; Yurkov 2018). The decomposition of lignocellulosic residues, such as straw and other organic matter sources, releases a complex combination of nutrients and inhibitory compounds, requiring microorganisms to develop specific resistance and tolerance mechanisms. Thus, isolating yeasts from these soils is a suitable strategy in order to select yeast strains capable of tolerating inhibitory compounds generated during the pretreatment of lignocellulosic biomass and utilizing renewable carbon sources for lipid production (Monteiro Moreira and Martins Do Vale 2020).

Oleaginous yeasts have been extensively investigated for lipid production from lignocellulosic substrates, particularly species such as *Yarrowia lipolytica* (Dias et al. 2024), *Rhodotorula toruloides* (Wankhede et al. 2025), and *Lipomyces starkeyi* (Zhang et al. 2021). However, lipid yields vary considerably among species and even among strains, especially regarding their capacity to metabolize pentoses and efficiently convert them into lipids (Brandenburg et al. 2021). In many cases, superior lipid production has been achieved using genetically engineered strains rather than wild-type isolates (Drzymała-Kapinos et al. 2022). Metabolic engineering strategies have therefore been employed to enhance xylose assimilation and improve tolerance to inhibitory compounds commonly present in lignocellulosic hydrolysates (Gallego-García et al. 2023).

Among non-conventional yeasts, species of the genus *Candida* have also been reported as oleaginous microorganisms capable of assimilating diverse carbon sources (Quarterman et al. 2018). However, most studies focus on defined media or purified substrates, and few have evaluated their performance in complex lignocellulosic hydrolysates containing inhibitory compounds (Vemparala et al. 2025; Mota et al. 2022). Similarly, research on brewing byproducts such as brewer’s spent grain hydrolysate as a substrate for *Candida* species remains scarce (Patel et al. 2018). Therefore, identifying robust strains capable of efficiently converting these complex substrates into lipids is crucial to improve the economic feasibility of microbial lipid production.

In this study, 61 yeast strains were isolated and characterized from soil samples, among which two *C. maltosa* strains, UFV-1 and UFV-2, were selected due to their ability to grow on xylose and accumulate more than 20% (w/w) lipids, even in the presence of acetic acid. Both strains grew in media containing lignocellulosic inhibitors, such as furfural, hydroxymethylfurfural, and formic acid, and also in non-detoxified malt bagasse hydrolysate, highlighting their metabolic robustness. Detoxification of the hydrolysate led to significant improvements in cell growth and lipid production. In a benchtop bioreactor cultivation with detoxified hydrolysate, *C. maltosa* UFV-1 showed a notable 4.7-fold increase in biomass titer compared to flask cultivations. This result underscores its potential to produce lipids from low-cost lignocellulosic feedstocks.

## Materials and methods

### Microorganisms, maintenance and inoculum preparation

The yeasts used in this work were isolated from soil samples from Unidade de Ensino, Pesquisa e Extensão em Produção de Grandes Culturas e Bioenergia (UEPE-GCBE Aeroporto), belonging to the Universidade Federal de Viçosa (UFV), Viçosa, Minas Gerais, Brazil. Yeasts were maintained in Yeast extract–peptone (YP) medium [% (w/v): 0.50 yeast extract, 0.50 peptone] with 30% (v/v) glycerol at -80 ºC (Kurtzman et al. 2011). The isolates are stored in the culture collection of the Laboratory of Microbial Physiology (LABFIS) of the Department of Microbiology from UFV. Before each experiment, yeasts were activated by transferring 0.20 mL stock into a 125 mL Erlenmeyer flask containing 30 mL of Yeast extract–peptone-dextrose (YPD) medium [% (w/v): 0.50 yeast extract, 0.50 peptone and 1.00 glucose] and incubated at 30 ºC and 200 rpm for 18 h (G25, New Brunswick, USA), as commonly used for yeast cultivation (Smith and Burke 2014). The suspension was centrifuged at 12,000 g for 10 min (Sigma D-37520, Osterode am Harz, Germany), and the cells were washed with 0.85% (w/v) NaCl. Before inoculation, the optical density at 600 nm (OD_600_), measured using a UV–visible spectrophotometer (BECKMAN DU series 600, Beckman Instruments Inc., USA), was adjusted to reach 0.1 in the beginning of each cultivation.

### Soil sampling and yeast isolation

Samples were collected from UEPE-GCBE Aeroporto (-20.748964, -2.842144), a station focused on agricultural research and the production of large crops and bioenergy. Composite samples were collected from three different points in three different collection areas, both at the surface (depth of 0 cm) and at a depth of 10 cm, with three portions taken at each point to ensure greater representation of soil conditions throughout the area analyzed.

Isolation was performed as described by Yurkov et al. 2016 and Kurtzman et al. 2011, with modifications, employing two strategies: direct plating and enrichment culture of environmental samples. For direct plating, one gram of composite soil sample was suspended in 9 mL of sterile 0.85% (w/v) NaCl solution (10⁻^1^ dilution) in a 15 mL tube and homogenized by vortex for 2 min. Serial dilutions (10^⁻1^ to 10^⁻6^) were prepared in sterile 0.85% (w/v) NaCl solution, and 100 µL aliquots were spread-plated onto solid Yeast extract–peptone-xylose (YPX) [% (w/v): 0.50 yeast extract, 0.50 peptone, 1.00 xylose] and Yeast nitrogen base-xylose (YNBX) [% (w/v): 0.67 yeast nitrogen base, 1.00 xylose] medium, both supplemented with 0.01% (w/v) chloramphenicol and 1.75% (w/v) agar. Spread plating was performed to ensure even distribution of cells. For the enrichment strategy, one gram of soil sample was inoculated into 50 mL of liquid YPX or YNBX medium in 125 mL Erlenmeyer flasks and incubated at 30 ºC, 200 rpm for 24 h. The culture was serially diluted (10^⁻1^ to 10^⁻6^) in sterile 0.85% (w/v) NaCl solution and plated onto their respective solid media (YPX-agar and YNBX-agar) using the same plating technique as for direct samples. The plates were incubated at 30 ºC and monitored daily during a 21-day incubation period, with macroscopic evaluations of colony morphology and microscopic evaluations of yeast cell structure to ensure accurate selection and purification of the isolates.

### Morphological characterization and grouping

The morphotypes of yeast cells and colonies were analyzed with at least one representative of each morphotype being purified and preserved for later identification. The purified colonies were cultivated on Yeast extract–malt extract–xylose (YMX) medium [% (w/v): 0.30 yeast extract, 0.50 peptone, 0.30 malt extract, 1.00 xylose] with 1.75% (w/v) agar, incubated at 30 °C for 48 h. Plates were then photographed to evaluate macroscopic characteristics (Canon EOS Rebel T7, EF-S 18–55 mm), and slides were prepared for microscopic analysis (BX50F4, 40x, Olympus Optical Co. Ltd.), following the procedure described by Kurtzman et al., (2011).

### PCR-fingerprinting

First, the genomic DNA of yeast isolates was extracted from pure cultures using phenol–chloroform-isoamyl alcohol method adapted from Bartlett and Stirling, (2003). Briefly, the isolates were first activated, cell lysis was performed by cryogenic disruption using liquid nitrogen, followed by mechanical agitation with glass beads in extraction buffer [50 mmol/L Tris–HCl, 150 mmol/L NaCl, 20 mmol/L EDTA, 10 mmol/L HCl, and 0.20% (w/v) SDS] for 3 min using a vortex. Proteins were precipitated with phenol:chloroform:isoamyl alcohol (25:24:1) and subsequently centrifuged for 10 min at 12,000 g. The resulting material was treated with 10 mg/mL RNAse (Sigma) and then precipitated in sodium acetate (3 M, pH 5.2) and 100% (v/v) ethanol overnight at -20 ºC. DNA samples were centrifuged and washed with 70% (v/v) ethanol. The DNA was rehydrated with 30 µL of deionized water at 60 °C for 1 h and stored at -20 °C.

PCR-fingerprinting analyses were performed with primers M13 (5′-GAGGGTGGCGGTTCT-3′) and (GAC)_5_ (5’-GAC GAC GAC GAC GAC-3’) (Andrade et al. 2006; Baleiras Couto et al. 1996). Primer M13 was initially used to generate banding patterns for all isolates. When isolates from the same location produced identical profiles, indicating the presence of genetically similar lineages, the complementary primer (GAC)_5_ was applied to achieve further discrimination between the isolates or to indicate that they represented the same original strain. Each PCR assay was carried out in a total volume of 25 μL containing: 1.0 μL input DNA, 1.0 μL of 10 mM dNTP (Promega), 4.0 μL of 25 mM MgCl_2_ (Promega), 1.5 μL of 10 μM primer (IDT), 1.0 μL 5 U *Taq* DNA Polymerase (Promega), 4.0 μL 5X PCR buffer (Promega) and sterile deionized water. PCR assays were performed in the PCR Express thermal cycler (Bio-Rad) as follows: initial denaturation at 94 °C for 2 min, followed by 40 cycles of 45 s of denaturation at 95 °C, 1 min of primer annealing at 50 °C and 1 min of extension at 72 °C, and a final extension for 6 min at 72 °C. PCR products were separated by 1.5% (w/v) agarose gel electrophoresis in 1.0X TBE buffer (1.11 M Tris-base, 0.1 M boric acid, 0.5 M EDTA, pH 8) for 180 min at 60 V, and visualized under UV light after staining with 0.05% (v/v) ethidium bromide (Sigma Aldrich, Milan, Italy) in a gel photodocumentation system (L-Pix). For band size comparison across gels, a control DNA sample from our isolates was included in every agarose gel run, allowing direct alignment and normalization of band patterns between different samples. The electrophoretic bands for each sample were hand marked and transformed into a binary matrix, which was then used for hierarchical grouping and represented as dendrograms using the “dendextend” R package (Galili 2015). Clustering and image analysis were conducted in R (v. 4.2.1).

### Screening of yeast isolates

#### First screening

Yeasts were activated and the cell suspensions were inoculated into 96-well microplates containing 200 µL of 0.67% (w/v) YNB medium supplemented with 2% (w/v) glucose or xylose in triplicate. Growth was monitored using the OD_600_ over 24 h for glucose and 48 h for xylose using a Multiskan GO microplate reader (ThermoScientific, Wilmington, DE, USA). Isolates that reached OD_600_ ≥ 0.5 in xylose were selected for further analysis. Growth in the presence of 2.0 g/L acetic acid was further assessed for the selected strains (triplicates). Isolates reaching OD_600_ ≥ 0.5 at the highest acetic acid concentration were selected for oleaginous phenotype screening.

#### Second screening

The yeasts selected in the first screening step were evaluated for their potential to produce lipids. The yeasts were inoculated into 125 mL Erlenmeyer flasks containing SS2 medium (Tanimura et al. 2014) 100:1 (C:N) [% (w/v): 0.010 yeast extract, 0.052 (NH_4_)_2_SO_4_, NaCl 0.010, CaCl_2_ 0.010, MgSO4 0.050] supplemented with 3% (w/v) xylose. This medium was used to promote lipid accumulation under nitrogen limitation, a condition known to trigger lipid biosynthesis in oleaginous yeasts (Chattopadhyay and Maiti 2021). In this formulation, (NH₄)₂SO₄ was the main nitrogen source used to define the C:N ratio, while yeast extract was maintained at a low fixed concentration solely as a source of growth factors. The experiment was performed in duplicates and cultivations were carried out for 96 h at 30 ºC and 200 rpm. Isolates presenting the oleaginous phenotype [lipid content ≥ 20% (w/w)] were selected and subsequently tested in the same medium supplemented with 1.0 g/L acetic acid, under the same conditions to assess its impact on lipid production. Biomass and lipid production were determined at the end of each cultivation. Cell dry weight (DW) was determined after freeze-drying and lipid quantification was conducted using the SPV (Sulfo-Phospho-Vanilin) method as described by Knight et al., (1972) with modifications. Both methods are detailed in Section "Analytical methods".

### D1/D2 sequencing

The best isolates selected in the second screening were subjected to taxonomic identification after differential grouping in the fingerprinting step initially by sequencing the D1/D2 domains of the gene encoding the 26S subunit of ribosomal DNA. The universal primers NL-1 (5′-GCATATCAATAAGCGGGAGGAAAAG-3′) and NL-4 (5′-GGTCCGTGTTTCAAGACGG-3′) were used for amplification of the D1/D2 region (Lachance et al. 1999). PCR was performed in a final volume of 50 μL, containing: 2.0 μL DNA, 1.0 μL of each primer (10 μmol) (IDT), 5.0 μL 5X delivery (Promega), 2.0 μL 25 mM MgCl₂ (Invitrogen), 2.0 μL 10 mM dNTP (Promega), 0.2 μL *Taq* DNA polymerase (5U) (Promega), and sterile deionized water. PCR was performed in a PCR Express thermocycler (Bio-Rad) with the following program: initial denaturation at 95 °C for 2 min, followed by 5 cycles of denaturation at 95 °C for 15 s, annealing at 54 °C for 25 s, and extension at 72 °C for 20 s, with a final extension at 72 °C for 10 min. PCR products were analyzed by 1.5% (w/v) agarose gel electrophoresis (Sigma Aldrich, Milan, Italy) in 1.0X TBE, for approximately 120 min at 80 V. Gels were stained with ethidium bromide (Sigma Aldrich, Milan, Italy), visualized under ultraviolet light, and photographed in a gel documentation system (L-Pix).

The PCR products were sequenced by Sanger method at BPI Biotechnology (São Paulo, Brasil). The obtained sequences were processed for taxonomic identification using the Geneious software (Version 2025.0.3, Dotmatics, Auckland, New Zealand) and the BLAST tool at NCBI (National Center for Biotechnology Information). After sequencing, raw files were imported into Geneious by creating a new folder and uploading the files corresponding to the forward and reverse reads. The sequences were aligned and assembled into contigs, with low-quality regions automatically trimmed during this process. Once the contigs were assembled, they were annotated by conducting a BLAST search against the NCBI nr database. For each sample, the best taxonomic match was identified based on the lowest e-value and higher sequence identity. The phylogenetic positioning of the isolates selected as potential lipid producers was represented by a tree constructed from the sequences of the D1/D2 region. The sequences used were obtained from different sources according to The Yeasts Database (https://theyeasts.org/species-search) and selected based on the highest genetic similarity. The sequences were aligned using the ClustalW algorithm (Thompson et al. 1994). The phylogenetic tree was constructed using the Maximum Likelihood method with the Kimura 2-parameter model, selected as the most appropriate evolutionary model based on log-likelihood. The bootstrap consensus tree was inferred from 1,000 replicates; branches reproduced in fewer than 50% of replicates were collapsed. The dataset included 13 nucleotide sequences with 570 positions in the final alignment. The rate variation model allowed for a proportion of evolutionary invariable sites. *Meyerozyma guilliermondi*i CBS 18143 was used as the outgroup to root the tree. Phylogenetic analysis was conducted in MEGA version 12 (Kumar et al. 2024) and the resulting tree was edited and visualized in the Interactive Tree Of Life (iTOL) platform (Letunic and Bork 2021).

### Impact of different carbon/nitrogen ratios on the oleaginous phenotype

The most promising oleaginous yeasts, that is, those that presented an oleaginous phenotype even in the presence of acetic acid in the previous screening step, were evaluated regarding lipid production under different carbon/nitrogen (C:N) ratios. The strains were inoculated into 125 mL Erlenmeyer flasks containing SS2 medium with varying C:N ratios (50:1, 75:1, 100:1, 125:1, and 150:1). Nitrogen concentrations were adjusted using (NH₄)₂SO₄ to final values of 0.109, 0.071, 0.052, and 0.041% (w/v), respectively, with a fixed concentration of xylose [3% (w/v)]. Cultivation was carried out at 30 °C and 200 rpm for 72 h in triplicates. At the end of cultivation, biomass and lipid production were determined as described in Section "Analytical methods". Results were analyzed using one-way ANOVA followed by Tukey’s post-hoc test to identify significant differences between treatments (*p* < 0.05).

### Growth in the presence of furfural, hydroxymethylfurfural and formic acid

The same strains assessed regarding the oleaginous phenotype in different C:N ratios were inoculated into 96-well microplates containing 200 µL of either 0.67% (w/v) YNB or YP medium (0.5% yeast extract, 0.5% peptone) with 2.0% (w/v) xylose. The media were supplemented with increasing concentrations of furfural (0.05; 0.10; 0.50; 1.00; 2.00 g/L), hydroxymethylfurfural (HMF) (0.10; 0.50; 1.00; 2.00; 4.00 g/L), and formic acid (0.20; 0.50; 1.00; 1.50; 2.00 g/L). The concentrations were selected to represent values found in lignocellulosic hydrolysates (Kumar et al. 2020; Vanmarcke et al. 2021). To assess whether the solvent used to dilute furfural had any effect on yeast growth, ethanol was added to the culture medium at the same concentration present in the highest furfural treatment (1.65 g/L, corresponding to furfural at 2.00 g/L). Cultivations were performed in quadruplicate at 30 ºC for 96 h. The OD_600_ was measured every 24 h using a Multiskan GO microplate reader (ThermoScientific, Wilmington, Delaware, USA).

### Preparation of malt bagasse hemicellulosic hydrolysate

The hemicellulosic hydrolysate obtained from malt bagasse was used to evaluate its applicability as a substrate for lipid production. The pretreatment was conducted by the Laboratory of Bioprocesses and Sustainable Products, Escola de Engenharia de Lorena, Universidade de São Paulo (USP). The biomass was initially air-dried, milled using a knife mill (Benedetti 270, Benedetti, Brazil), and sieved to reach an average particle size of 4.7 mm. Acid pretreatment was carried out using diluted H₂SO₄ (Vetec®) in 200 mL stainless steel reactors operating as a closed system and maintained in a thermostatic water bath. The pretreatment conditions were 2% (v/v) sulfuric acid (H_2_SO_4_, Vetec®), 165 °C, and 40 min of reaction. After pretreatment, the hydrolysates were cooled, filtered, and stored at 4 °C until further use Mesquita et al. 2016). Proteins and other particles in suspension were removed from the hydrolysate before quantifying sugar and inhibitors, following the precipitation protocol of Siegfried et al. (1984). Glucose, xylose, arabinose, acetic acid, formic acid, furfural, and HMF were determined by High-Performance Liquid Chromatography (HPLC) performed by the authors, using a RID-20A refractive index detector (Shimadzu, Japan) and an Aminex HPX-87H column (300 × 7.8 mm) (Bio-Rad, USA) at 45 °C, with 5 mM sulfuric acid as the mobile phase and a flow rate of 0.7 mL/min.

### Preparation of detoxified and non-detoxified hemicellulosic hydrolysate

The hydrolysate was prepared in both detoxified and non-detoxified forms to evaluate their effects on microbial growth and lipid production (Section "Effect of pH and nitrogen source on yeast growth and lipid production in malt bagasse hemicellulosic hydrolysate" and "Batch bioreactor cultivation of C. maltosa UFV-1 using detoxified malt bagasse hemicellulosic hydrolysate"). For the preparation of non-detoxified hydrolysate, the pH was first adjusted to the target cultivation (see Section "Effect of pH and nitrogen source on yeast growth and lipid production in malt bagasse hemicellulosic hydrolysate") value using sodium hydroxide (NaOH) beads under constant stirring, followed by centrifugation at 4000 g for 10 min. The supernatant was vacuum filtered using Whatman filter paper, supplemented with nitrogen sources (see 2.11), then filtered using a 0.22 µm membrane and stored at 4 °C until use.

The hydrolysate detoxification process was carried out as described by Marton et al. 2006, with adaptations. The detoxification process began with pH adjustment to 7.0 using calcium oxide (CaO) under continuous agitation, followed by centrifugation at 3000 g for 15 min. The supernatant was then acidified to pH 2.5 with phosphoric acid (H₃PO₄) and treated with activated charcoal 1% (w/v) at 60 °C and 150 rpm for 30 min. After vacuum filtration through a Whatman paper, the hydrolysate underwent the final preparation steps: readjustment of the pH to the target culture value (see Section "Effect of pH and nitrogen source on yeast growth and lipid production in malt bagasse hemicellulosic hydrolysate" and "Batch bioreactor cultivation of C. maltosa UFV-1 using detoxified malt bagasse hemicellulosic hydrolysate") using NaOH beads, centrifugation at 4000 g for 10 min, supplementation with nitrogen sources (see Section "Effect of pH and nitrogen source on yeast growth and lipid production in malt bagasse hemicellulosic hydrolysate" and "Batch bioreactor cultivation of C. maltosa UFV-1 using detoxified malt bagasse hemicellulosic hydrolysate"), filtration through a 0.22 µm membrane, and storage at 4 °C.

### Effect of pH and nitrogen source on yeast growth and lipid production in malt bagasse hemicellulosic hydrolysate

To investigate the influence of pH and nitrogen source on yeast growth and lipid production in malt bagasse hemicellulosic hydrolysate, the following experimental strategy was employed: (i) preliminary evaluation of the effects of pH and nitrogen source in non-detoxified hydrolysate; (ii) optimization step performed through a Central Composite Rotational Design (CCRD) targeting growth and lipid production in non-detoxified hydrolysate; and (iii) validation of the conditions predicted by the model using non-detoxified and detoxified media. The optimization of pH and nitrogen source was prioritized due to the intrinsic characteristics of the hemicellulosic malt bagasse hydrolysate obtained by acid hydrolysis, which typically presents very low pH (often < 1) and limited availability of assimilable nitrogen. These factors directly affect yeast growth and lipid accumulation through their influence on inhibitor toxicity and C:N balance (Beopoulos et al. 2009). In this stage, peptone and yeast extract were used as nutritional supplements to support growth in the complex hydrolysate matrix.

In the preliminary evaluation, yeasts were cultivated in 96-well microplates, incubated at 30 ºC for 50 h under agitation using a Multiskan GO microplate reader (ThermoScientific, Wilmington, DE, USA). Cultivations were carried out in non-detoxified hydrolysate to evaluate the effects of pH and nitrogen source on cell growth with pH values adjusted to 2.0, 3.0, 4.0, 5.0, 6.0 and 7.0 using NaOH, and two nitrogen sources (peptone or yeast extract, both at 1.0 g/L). These experiments were conducted with four biological replicates.

Based on the results obtained in the microplate cultivations, a Central Composite Rotational Design (CCRD) with response surface methodology (RSM) was applied to determine the optimal initial pH and peptone:yeast extract ratio – P:YE, with growth and lipid production as response variables. The experiment comprised 11 samples for each yeast, with 3 replicates at the central point (Table 1). The independent variables were evaluated at five levels (− α, − 1, 0, + 1, + α), allowing the estimation of linear, quadratic, and interaction effects. Cultivations were conducted in 250 mL Erlenmeyer flasks containing 75 mL of medium and incubated at 30 °C and 200 rpm for 60 h. The dependent variables analyzed during optimization were specific growth rate (µ; h-1), final biomass (g/L), lipid content [% (w/w)] and lipid titer (g/L). Data was first analyzed using an ANOVA to evaluate the significance of the main effects and interactions. A predictive mathematical model was fitted to the data based on a second-order polynomial equation, according to Eq. 1.

1 $$y={\beta }_{0}+\sum {\beta }_{i}+{X}_{i}+\sum {\beta }_{ii}+{X}_{{i}^{2}}+\sum {\beta }_{ij}+{X}_{i}{X}_{j}$$

where y represents the response variable, that is, growth rate (µ; h-1), final biomass (g/L), lipid content [% (w/w)] or lipid titer (g/L); ${X}_{i}$ and ${X}_{j}$ are the independent variables (pH and the P:YE), and ${\beta }_{0}$,${\beta }_{i}$, ${\beta }_{ii}$ and ${\beta }_{ij}$ are the estimated model coefficients. The quality of the fit was evaluated using the coefficient of determination (R^2^) and the significance of the model terms. Response surface plots and contour graphs were generated to visualize the optimal cultivation regions.

**Table 1:** Experimental range and levels of independent variables used in the Central Composite Rotational Design (CCRD)

Independent variables	Range and levels				
	− α	− 1	0	+ 1	+ α
Initial pH	6.0	6.1	6.5	6.9	7.0
P:YE	1:0	0.7:0.3	0.5:0.5	0.3:0.7	0:1

After defining the optimal cultivation conditions, the predicted model was validated using detoxified and non-detoxified hydrolysates, applying the same parameters used during the optimization step (30 °C, 200 rpm, 75 mL) for 60 h. Samples were collected at the beginning and end of cultivation and subjected to a protein precipitation protocol described by Siegfried et al. (1984) prior to HPLC analysis. Glucose, xylose, arabinose, acetic acid, formic acid, furfural, HMF, glycerol, and ethanol were then quantified as described in Section "Preparation of malt bagasse hemicellulosic hydrolysate". At the end of cultivation, biomass was also collected for lipid quantification and fatty acid profile characterization.

### Batch bioreactor cultivation of *C. maltosa* UFV-1 using detoxified malt bagasse hemicellulosic hydrolysate

The cultivations were carried out in duplicate in 1.3 L bench-top bioreactors (BioFlo/CelliGen 115, Eppendorf, Germany) containing 900 mL of detoxified hemicellulosic malt bagasse hydrolysate supplemented with 1 g/L peptone and 200 mg/L chloramphenicol. The addition of the antibiotic aimed to prevent bacterial growth arising from residual spores that might persist in the hydrolysate after the filtration process. Operational conditions were maintained at 400 rpm, with the addition of Antifoam 204 (Sigma-Aldrich) at a final concentration of 400 µL/L. Unlike the other experiments, the cultivation temperature was set to 31 °C, since preliminary assays indicated higher growth rate and biomass accumulation by *C. maltosa* UFV-1 under this condition. The initial volumetric oxygen transfer coefficient (kLa), previously determined by the nitrogen gassing-out method (Garcia-Ochoa and Gomez 2010) based on dissolved oxygen concentration measurements, was 93.03 h⁻^1^. No cascade control strategy was employed to maintain the dissolved oxygen concentration. The initial optical density (OD₆₀₀) was adjusted to 0.2, and the total cultivation time was 48 h. At the end of cultivation, the biomass was harvested and used for lipid quantification.

### Analytical methods

#### Specific growth rate

Specific growth rate (µ; h^−1^) was calculated by applying a linear regression to the ln (OD_600_) readings plotted against time (h) during the exponential growth phase of cell growth.

#### Dry biomass

At the end of cultivation, 40 mL of the medium was centrifuged at 4,000 g for 10 min. The pellet was washed twice with a 0.85% (w/v) NaCl solution. After washing, the biomass was freeze-dried for 24 h to ensure complete dehydration. The dry biomass was then determined gravimetrically, and the concentration calculated in g/L based on the initial volume of the culture medium.

#### Lipid quantification

Lipids were quantified using the SPV method as described by Knight et al., (1972) with adaptations. The phosphor-vanillin (PV) reagent was freshly prepared by dissolving 600 mg of vanillin into 10 mL of absolute ethanol, subsequently diluted to 500 mL with 90 mL of water and 400 mL of 85% (w/v) phosphoric acid. For the standard curve, commercial soybean oil was diluted in methanol:chloroform 2:1 to a final concentration of 1.5 mg/mL; the curve comprised a lipid mass ranging from 50–250 µg. For quantification, cell dry biomass (10 mg) was diluted in 2 mL of water. Then, the 100 µL of the biomass dilution was mixed with concentrated sulfuric acid (2 mL) and incubated at 100 °C for 10 min. After cooling to room temperature (20 °C), 2 mL of the PV reagent was added for the colorimetric reaction. The mixture was incubated at 37 °C for 15 min, then transferred to 96-well flat-bottom microplates for absorbance measurements. Absorbance was measured at 530 nm using a Multiskan FC plate reader (Thermo Scientific, San Jose, CA, USA).

#### Fatty acid profile

Fatty acid profile analysis was performed according to the Sherlock Microbial Identification System protocol (version 6.0, Microbial ID, Inc., Newark, DE, USA). For fatty acid extraction, 4–5 mg of freeze-dried biomass was subjected to saponification and methylation steps to produce fatty acid methyl esters (FAMEs). FAMEs were extracted and analyzed using a gas chromatograph (Agilent 7890A, Agilent Technologies, Santa Clara, CA, USA) equipped with a fused silica capillary column coated with 5% phenylmethyl silicone and a flame ionization detector (FID), following the default configuration of the Sherlock system. Fatty acids were identified by comparison with the RTSBA6 library using Sherlock MIDI software.

#### HPLC analysis

The concentration of xylose (0.75 to 30.0 g/L), glucose (0.75 to 30.0 g/L), arabinose (0.15 to 6.00 g/L), acetic acid (0.05 to 6.00 g/L), formic acid (0.01 to 6.00 g/L), furfural (0.02 to 0.80 g/L), HMF (0.02 to 0.80 g/L), glycerol (0.13 to 5.00 g/L) and ethanol (0.13 to 5.00 g/L) was determined by HPLC as follows: LC-20AT HPLC system (Shimadzu, Japan) coupled to a RID-20A refractive index detector (Shimadzu, Japan) and an Aminex HPX-87H ion exchange column (300 × 7.8 mm, 9 μm, Bio-Rad, Munich, Germany). The mobile phase used was 5 mM H_2_SO_4_ with a flow rate of 0.7 mL/min at 45 °C.

#### Determination of lipid production parameters

The lipid production parameters were determined as follows:

2 $$Lipid content (\% w/w) = (P/DW)\times 100$$

where $P$ is final lipids (mg) (see 2.2.11.3); DW is cell dry weight (mg).

3 $$Lipid titer (g/L)=Lipid content (\% w/w) \times {X}_{f}$$

where ${X}_{f}$ is final biomass (g/L) (see 2.2.11.2).

4 $$Lipid Productivity (g/L h)=\frac{Lipid Titer (g/L)}{t}$$

where t is the total cultivation time (h).

5 $$Lipid Yield ({Y}_{P/s})=\frac{Lipid titer (g/L)}{{S}_{0}-{S}_{f}}$$

where ${S}_{f}$ ≡ final sugar concentration (g/L); ${S}_{0}$ ≡ initial sugar concentration (g/L). The sugar concentration includes the sum of glucose, xylose and arabinose in cultivations with hemicellulosic hydrolysates and pure sugars in defined media during the screening steps.

## Results

### Isolation and PCR-fingerprinting

Sixty-one yeasts were isolated from soil samples collected at the UEPE-GCBE Aeroporto. The isolates were identified using the following nomenclature system: “C” indicates the collection of samples from locations near sugarcane plantations, followed by “P” (depth) or “S” (surface), then “I, II or III” (corresponding to different regions within the sampling site) and finally, the number represents the order of isolation. After morphological characterization and grouping, which included the evaluation of macro- and microscopic features, and considering the maintenance of viability through successive subcultures, the number of isolates was reduced to 31.

**Fig. 1:**
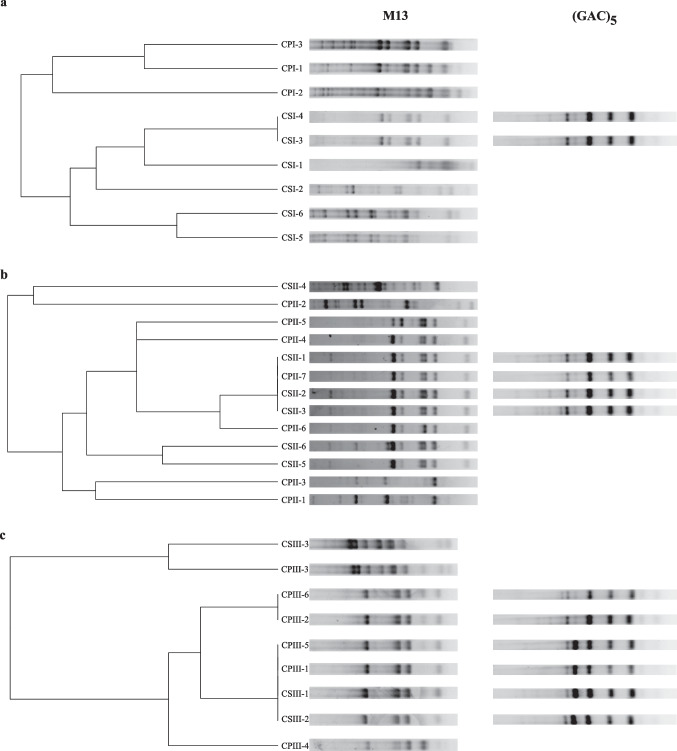
Dendrogram generated from PCR-fingerprinting profiles obtained with primers M13 and (GAC)_5_ for 31 yeast isolates from three different locations (I—a, II—b, and III—c) at UEPE-GCBE Aeroporto. The dendrogram was constructed based on the profiles obtained with the M13 primer. Isolates that showed identical profiles with the M13 primer were subsequently amplified with the (GAC)_5_ primer for possible distinction among genetically similar strains

The PCR-fingerprinting using primers M13 and (GAC)5 was performed to assess the genetic relatedness among the 31 yeast isolates. Comparisons were conducted only within each sampling location (I, II, and III; Fig. 1a, b, and c, respectively), without cross-comparison between different areas. The number of genetically distinct isolates was reduced from 9 to 8 at site A, from 13 to 10 at site B, and from 9 to 4 at site C, resulting in a final set of 22 distinct yeast isolates (Fig. 1).

### Screening of yeast isolates capable of growing on sugars constituents of lignocellulosic biomasses and tolerating acetic acid

**Fig. 2:**
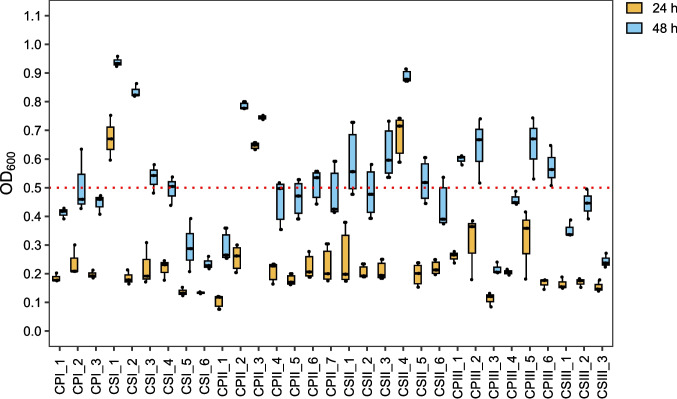
Final OD_600_ of yeasts isolated from three different locations isolated at UEPE-GCBE Airport after 24 (yellow) and 48 (blue) hours of cultivation in YNB medium with xylose (20 g/L). The dotted red line (OD₆₀₀ ≥ 0.5) indicates the cutoff point used for selection of isolates based on the growth observed at 48 h. The boxplots represent the median (horizontal line), interquartile range (box) and individual values ​​(black dots)

The 31 isolates identified after the evaluation of macro- and microscopic features were initially cultivated in 96-well microplates with YNB medium containing 20 g/L glucose as the sole carbon source for 24 h. We observed variations in their growth performance (Fig. S1). Subsequently, these isolates were grown in medium with xylose (20 g/L) to identify the strains that displayed the better capacity of growth from this pentose, as it is the major constituent of the hemicellulosic fraction of lignocellulosic biomasses. A cutoff point of OD600 ≥ 0.5 at 48 h was used as the selection criterion, resulting in the selection of 16 isolates (Fig. 2).

**Fig. 3:**
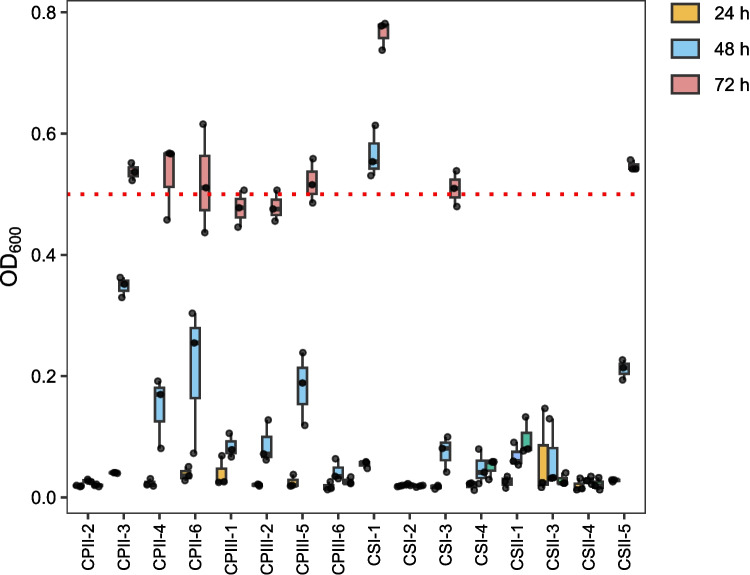
OD_600_ of yeasts isolated from three different locations isolated at UEPE-GCBE Airport after 24 (yellow), 48 (blue), and 72 (red) h of cultivation in YNB medium supplemented with 20 g/L xylose and 2 g/L acetic acid. The red dashed line (OD_600_ ≥ 0.5) indicates the threshold used to select isolates based on their growth at 72 h. Boxplots represent the median (horizontal line), interquartile range (box), and individual values (black dots)

The 16 selected isolates that presented OD_600_ ≥ 0.5 in YNB medium plus xylose had their tolerance to acetic acid evaluated in YNB medium with 20 g/L of this pentose and 2 g/L of this acid. The OD_600_ measurements were taken after 24, 48, and 72 h (Fig. 3). In this step, an OD_600_ ≥ 0.5 after 72 h of cultivation was used as the selection criterion. Isolates CPII-3, CPII-4, CPII-6, CPIII-5, CSI-1, CSI-3, and CSII-5 were selected for further analysis.

### Screening of selected yeast isolates regarding lipid production

**Fig. 4:**
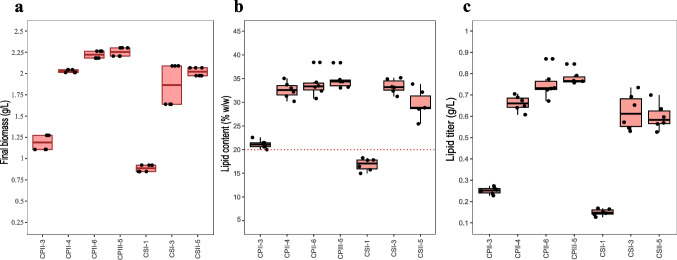
**a** Final biomass (g/L), **b** lipid content [% (w/w)], and **c** lipid titer (g/L) of yeast strains grown in SS2 medium (C:N 100:1). Boxplots show the median (line), interquartile range (box), and individual values (black dots). The red dotted line in **b** indicates the 20% (w/w) lipid content threshold

In order to evaluate the lipid production, the 7 yeasts selected in the previous step were cultivated in SS2 medium with a C:N of 100:1 (Fig. 4). Isolates CPII-4, CPII-6, CPIII-5, CSI-3 and CSII-5 reached a final biomass close to 2.0 g/L, while CPII-3 and CSI-1 recorded dry biomass values below 1.5 g/L (Fig. 4a). As a selection criterion, we adopted the lipid content of 20% (w/w), which is considered for classifying microorganisms as oleaginous. We verified that the isolates CPII-4, CPII-6, CPIII-5, CSI-3, and CSII-5 displayed lipid titers above 20% (w/w) of their DW (Fig. 4b). Based on the highest lipid contents, five isolates were selected for the next stage: CPII-3, CPII-4, CPII-6, CPIII-5, and CSII-5.

**Fig. 5:**
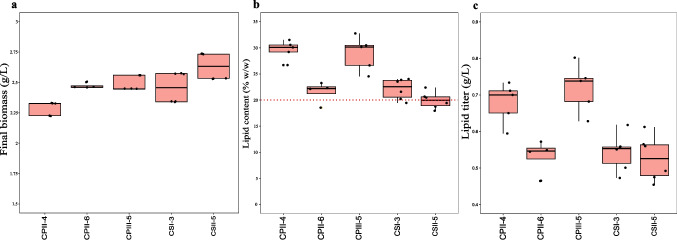
**a** Final biomass (g/L), **b** lipid content [% (w/w)], and **c** lipid titer (g/L) of yeast strains grown in SS2 medium (C:N 100:1) supplemented with 1.0 g/L acetic acid. Boxplots show the median (line), interquartile range (box), and individual values (black dots). The red dotted line in **b** indicates the 20% (w/w) lipid content threshold

To evaluate the impact of acetic acid on lipid production, CPII-3, CPII-4, CPII-6, CPIII-5, and CSII-5 were grown in SS2 medium (C:N 100:1) supplemented with 1.0 g/L of this acid (Fig. 5). CPII-6, CSI-3 and CSII-5 had the highest decreases in lipid content compared to the cultivation without acetic acid (Figs. 5b and 4b). Otherwise, CPII-4 and CPIII-5 were less affected by the presence of the acid, presenting high biomass and lipid titers (Fig. 5a and c).

### Taxonomic identification of isolates CPII-4 and CPIII-5

**Fig. 6:**
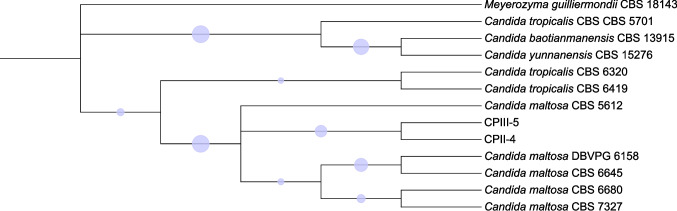
Phylogenetic placement of *C. maltosa* UFV-1 (CPII-4) and UFV-2 (CPIII-5) strains based on the alignment of the D1/D2 domain of the 26S rRNA gene. The tree was constructed using the Maximum Likelihood method based on the Kimura two-parameter model of sequence evolution. *Meyerozyma guilliermondii* CBS 18143 was used as the outgroup. Branch support is indicated by circles, with the size of each circle proportional to the bootstrap value (1000 replicates). The lowest bootstrap value shown is 0.55, and the highest is 1.00

The D1/D2 region of the ribosomal DNA allowed the identification of isolates CPII-4 and CPIII-5 as *Candida maltosa*, here referred to as strains UFV-1 and UFV-2, respectively. The phylogenetic positioning of the two strains constructed based on the D1/D2 region sequence also corroborated the identification of these two strains as *C. maltosa* (Fig. 6).

### Effect of different C:N ratios on the oleaginous phenotype of *C. maltosa* UFV-1 and UFV-2

**Fig. 7:**
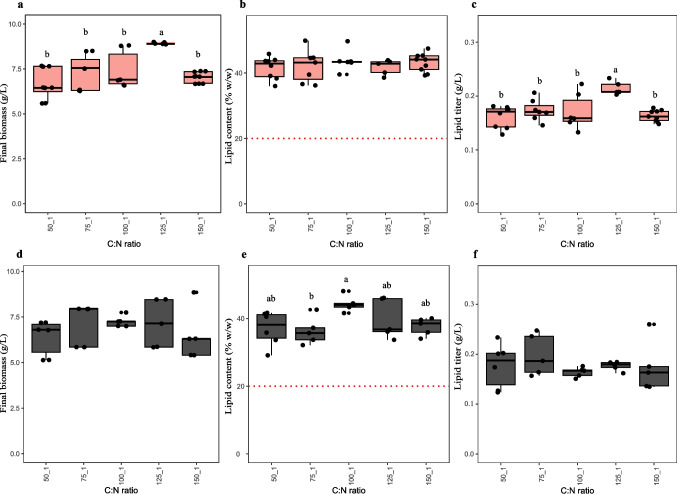
Final biomass (**a**, **d**), lipid content (**b**, **e**), and lipid titer (**c**, **f**) of *C. maltosa* UFV-1 (CPII-4; a–c; pink) and UFV-2 (CPIII-5; d–f; gray) cultivated in SS2 medium with different C:N ratios. Boxplots represent the median (horizontal line), the interquartile range (box), and individual data points (black dots). ANOVA was performed separately for each variable. When significant differences were found (*p* < 0.05), a Tukey’s post-hoc test was applied. Different letters above the boxplots indicate statistically significant differences between groups (*p* < 0.05). The red dotted line in panel B represents the 20% (w/w) lipid content threshold

Both *C. maltosa* UFV-1 and UFV-2 were cultivated under five different C:N ratios (50:1, 75:1, 100:1, 125:1, and 150:1) to evaluate the effect of nitrogen availability on lipid production (Fig. 7). For *C. maltosa* UFV-1, we did not observe significant differences for lipid content (*p* > 0.05; Fig. 7b); meanwhile the biomass and lipid titer were significantly higher (*p* < 0.05) at the 125:1 ratio (Fig. 7a). For *C. maltosa* UFV-2, only the lipid content significantly varied (*p* < 0.05), with the highest value at 100:1 and the lowest at 75:1 (Fig. 7e).

### Effects of furfural, HMF and formic acid on *C. maltosa* UFV-1 and UFV-2 growth

Acetic acid stands out as the main inhibitor found in hemicellulosic hydrolysates, even after detoxification steps; nevertheless, other inhibitors such as furfural, HMF, and formic acid also impair yeast growth. Thus, the effects of these inhibitors, which were not evaluated in the screening steps, on the growth of both *C. maltosa* UFV-1 and UFV-2 were evaluated in minimal (YNB) and complex (YP) media supplemented with xylose using different concentrations of each compound (Fig. S2). Both strains showed growth in media with furfural and HMF, achieving final OD_600_ values similar to the control (0 g/L) at concentrations up to 2.0 and 4.0 g/L, respectively, in both media. Formic acid was the most inhibitory compound, reducing the final OD_600_ values from 0.5 g/L onward. As the formic acid concentrations increased, the OD_600_ in YP was slightly higher than in YNB.

### Definition of cultivation conditions for *C. maltosa* UFV-1 and UFV-2 in malt bagasse hemicellulosic hydrolysate

To evaluate the growth of both *C. maltosa* strains (UFV-1 and UFV-2) in malt bagasse hemicellulosic hydrolysate, different pH values (2.0 to 7.0) and two nitrogen sources (yeast extract or peptone; 1.0 g/L) were tested (Fig. S3). We did not observe growth at pH 2.0 and 3.0 for both strains. On the other hand, they grew well at pH 6.0 and 7.0, reaching OD_600_ close to 1.7 in microwell assays, regardless of the nitrogen source. We also observed that the growth was slightly lower at pH 5.0. At pH 4.0, only *C. maltosa* UFV-1 grew in the presence of peptone; however, we verified a long lag phase of approximately 35 h, reaching a maximum OD_600_ of 0,95 (Fig. S3).

**Fig. 8:**
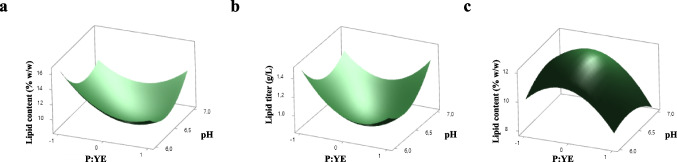
Response surface plots generated from statistically significant quadratic models obtained by Central Composite Rotational Design (CCRD). The surfaces represent model-predicted responses for the effects of initial pH and peptone:yeast extract (P:YE) ratio on lipid production by *C. maltosa* UFV-1 (**a** and **b**) and UFV-2 (**c**). Panels A and C show lipid content (% w/w), while panel B represents lipid titer (g/L)

To enhance both biomass and lipid production by *C. maltosa* strains in malt bagasse hemicellulosic hydrolysate, we conducted a CCRD based on the preliminary tests evaluating the effects of initial pH and nitrogen source (Table 2). For *C. maltosa* UFV-1, we obtained statistically significant models (*p* < 0.05) for lipid content and titer (R^2^ values of 85.07 and 81.72%; adjusted R^2^ of 70.14 and 63.44%, respectively). Both quadratic terms and the interaction between initial pH and P:YE ratio significantly influenced the responses. Even though a true optimum point was not reached, a condition of maximum lipid production was identified, within model boundaries, at initial pH 6.0 and a P:YE ratio of 1:0 g/L (coded − 1), with predicted values of 16.43% lipid content and 1.485 g/L lipid titer (Fig. 8). The fitted equations are shown in Eq. 6 and Eq. 7.

**Table 2:** Central Composite Rotational Design (CCRD) for optimization of initial pH and peptone:yeast extract ratio on yeast growth and lipid production in *C. maltosa* UFV-1 and UFV-2

Treatment	Initial pH	Peptone:Yeast extract (g/L)	Specific grow rate (h^−1^)	Final biomass (g/L)	Lipid content (% w/w)	Lipid titer (g/L)
*C. maltosa* UFV-1						
1	6.0	0.5:0.5	0.283	9.93	12.61	1.251
2	6.1	0.7:0.3	0.260	9.30	11.99	1.115
3	6.1	0.3:0.7	0.221	9.86	11.07	1.091
4	6.5	1:0	0.221	8.58	12.61	1.082
5	6.5	0:1	0.262	10.09	10.01	1.010
6	6.9	0.7:0.3	0.269	9.23	9.05	0.835
7	6.9	0.3:0.7	0.258	10.20	8.98	0.916
8	7.0	0.5:0.5	0.284	10.05	8.26	0.831
9*	6.5	0.5:0.5	0.260	10.52	9.60	1.010
9*	6.5	0.5:0.5	0.249	10.57	11.31	1.196
9*	6.5	0.5:0.5	0.248	9.91	9.82	0.974
*C. maltosa* UFV-2						
1	6.0	0.5:0.5	0.290	10.16	10.97	1.114
2	6.1	0.7:0.3	0.295	10.48	11.38	1.193
3	6.1	0.3:0.7	0.328	10.18	10.73	1.093
4	6.5	1:0	0.273	9.47	10.80	1.023
5	6.5	0:1	0.283	9.55	11.86	1.132
6	6.9	0.7:0.3	0.289	8.91	11.96	1.066
7	6.9	0.3:0.7	0.250	10.43	11.27	1.174
8	7.0	0.5:0.5	0.300	10.95	12.20	1.336
9*	6.5	0.5:0.5	0.273	9.60	10.35	0.993
9*	6.5	0.5:0.5	0.380	10.33	9.05	0.935
9*	6.5	0.5:0.5	0.287	9.28	9.14	0.848

*central point

6 $$\begin{aligned} &Lipid content=8.770-0.775P:YE-0.361pH\\&+1.170P:{YE}^{2}+1.19{pH}^{2}+0.658P:YE\times pH \end{aligned}$$

7 $$\begin{aligned} &Lipid titer = 0.860-0.049P:YE+0.0076pH\\&+0.132P:{YE}^{2}+0.099{pH}^{2}+0.052P:YE\times pH \end{aligned}$$

For *C. maltosa* UFV-2, only the lipid content model was significant (*p* < 0.05) (R^2^ = 79.61%; adjusted R^2^ = 59.23%). The predicted optimum (P:YE = 0:58:042 g/L; pH = 6.5) corresponded to a lipid content of 11.95% (Fig. 8). The regression model is shown in Eq. 8.

8 $$\begin{aligned} &Lipid content=11.81-0.716P:YE-0.056pH\\&-0.900P:{YE}^{2}-0.340{pH}^{2}-0.162P:YE\times pH \end{aligned}$$

The response surface plots indicated distinct optimization behaviors between the strains. While UFV-1 did not show a clear maximum within the tested range—suggesting potential for further improvement under different conditions—*C. maltosa* UFV-2 exhibited a well-defined optimum (Fig. 8). The models for specific growth rate and final biomass were not statistically significant (*p* > 0.05) for both strains. Despite the lack of significant models, the experimental data showed that both strains were able to grow well in malt bagasse hydrolysate, with specific growth rates ranging from 0.221 to 0.284 h⁻^1^ and final biomass values between 8.58 and 10.52 g/L for *C. maltosa* UFV-1, and 0.238 to 0.330 h⁻^1^ and 9.28 to 10.48 g/L for *C. maltosa* UFV-2.

The validation of the maximum conditions was first carried out using non-detoxified hemicellulosic malt bagasse hydrolysate. The experimental values were below those predicted by the fitted models: UFV-1 reached 9.21 ± 1.05% lipids and 0.84 ± 0.10 g/L lipid titer (predicted: 16.43% and 1.485 g/L, respectively), while UFV-2 achieved 8.74 ± 0.77% lipids (predicted: 11.95%) (Table 3). Meanwhile, a further step of validation was carried out with detoxified hydrolysate. All physiological parameters improved for both strains, except sugar consumption in UFV-2, which showed no significant difference. For UFV-1, significant increases (*p* < 0.05) were observed for biomass, sugar consumed, lipid content, lipid titer, productivity and lipid yield, reaching 17.0 g/L, 53.2 g/L, 18.2%, 3.14 g/L, 0.05 g/L·h and 0.06 g/g, respectively. Similarly, UFV-2 showed significant improvements in the same parameters (12.9 g/L, 45.6 g/L, 12.2%, 1.58 g/L, 0.03 g/L·h, and 0.03 g/g, respectively).

**Table 3:** Physiological parameters of *C. maltosa* UFV-1 and UFV-2 after 60 h of cultivation in non-detoxified and detoxified malt bagasse hemicellulosic hydrolysates under optimized conditions. Comparisons between non-detoxified and detoxified conditions were performed separately for each strain using Tukey’s test (*p* < 0.05). Values represent mean ± standard deviations. Different letters within the same strain indicate significant differences

Parameters	*Candida maltosa* UFV-1		*Candida maltosa* UFV-2	
	Non-detoxified	Detoxified	Non-detoxified	Detoxified
Final Biomass (g/L)	9.12 ± 0.10 b	17.0 ± 1.42 a	10.5 ± 0.11 b	12.9 ± 0.33 a
Sugar Consumed (g/L)	43.0 ± 3.36 b	53.2 ± 1.03 a	43.1 ± 2.41 a	45.6 ± 0.57 a
Lipid content (% w/w)	9.21 ± 1.05 b	18.2 ± 2.96 a	8.74 ± 0.77 b	12.2 ± 1.43 a
Lipid Titer (g/L)	0.84 ± 0.10 b	3.14 ± 0.76 a	0.91 ± 0.08 b	1.58 ± 0.19 a
Lipid Productivity (g/L h)	0.01 ± 0.00 b	0.05 ± 0.01 a	0.02 ± 0.00 b	0.03 ± 0.01 a
Lipid Yield ${(Y}_{p/s})$	0.02 ± 0.00 b	0.06 ± 0.01 a	0.02 ± 0.00 b	0.03 ± 0.00 a

Notably, glucose was completely consumed by both *C. maltosa* strains under all evaluated conditions (Table 4). The xylose assimilation was more efficient in the detoxified hydrolysate, especially by *C. maltosa* UFV-1, with concentrations decreasing from 12.3 ± 0.30 g/L to 0.45 ± 0.09 g/L. Arabinose was not consumed in the non-detoxified hydrolysate; otherwise, its concentration decreased in the detoxified medium for both strains. Inhibitory compounds such as furfural and HMF were reduced after detoxification and were almost completely metabolized by *C. maltosa* strains after 60 h of cultivation in both media. In contrast, the concentrations of organic acids (acetic and formic) were not affected by detoxification. Both organic acids were also almost completely consumed in the non-detoxified hydrolysate by both strains. However, in detoxified medium, only formic acid was fully metabolized by both strains, while acetic acid remained unconsumed in the culture with UFV-2. Ethanol was observed, exceeding 16 g/L in all conditions, with the highest values in cultivations with non-detoxified hydrolysate. Glycerol was also produced and showed slightly higher concentrations in non-detoxified media, especially for UFV-1. It should be noted that these cultivations were conducted in flasks, that is, under oxygen-limiting conditions, which favor fermentative metabolism.

**Table 4:** Characterization of non-detoxified and detoxified malt bagasse lignocellulosic hydrolysate before and after 60 h of cultivation during the validation of the CCRD model with *C. maltosa* UFV-1 and UFV-2. Values represent means concentrations ± standard deviations

Compound (g/L)	Non-detoxified		Detoxified	
	Initial	Final	Initial	Final
*C. maltosa* UFV-1				
Glucose	37.80 ± 1.75	0.00	39.60 ± 0.46	0.00
Xylose	11.30 ± 0.66	6.14 ± 1.04	12.30 ± 0.30	0.45 ± 0.09
Arabinose	4.21 ± 0.42	4.21 ± 0.42	4.23 ± 0.07	2.49 ± 0.43
Acetic acid	0.80 ± 0.03	0.06 ± 0.01	0.93 ± 0.02	0.09 ± 0.05
Formic acid	0.21 ± 0.01	0.06 ± 0.00	0.20 ± 0.02	0.08 ± 0.00
Furfural	0.30 ± 0.01	0.00	0.26 ± 0.01	0.00
HMF	0.69 ± 0.05	0.11 ± 0.02	0.55 ± 0.01	0.13 ± 0.02
Glycerol	0.00	1.67 ± 0.24	0.00	0.49 ± 0.17
Ethanol	0.00	20.50 ± 0.88	0	16.40 ± 1.74
*C. maltosa* UFV-2				
Glucose	37.80 ± 1.75	0.00	39.60 ± 0.46	0.00
Xylose	11.30 ± 0.66	5.95 ± 0.28	12.30 ± 0.30	4.57 ± 0.30
Arabinose	4.21 ± 0.42	4.21 ± 0.42	4.22 ± 0.07	2.96 ± 0.09
Acetic acid	0.80 ± 0.03	0.06 ± 0.00	0.93 ± 0.02	1.06 ± 0.06
Formic acid	0.22 ± 0.01	0.06 ± 0.00	0.20 ± 0.02	0.04 ± 0.00
Furfural	0.30 ± 0.01	0.00	0.26 ± 0.01	0.00
HMF	0.69 ± 0.05	0.18 ± 0.05	0.55 ± 0.01	0.11 ± 0.00
Glycerol	0.00	1.37 ± 0.03	0.00	0.77 ± 0.19
Ethanol	0.00	19.3 ± 1.13	0.00	16.2 ± 2.79

The fatty acid profile of *C. maltosa* UFV-1 and UFV-2 varied between non-detoxified and detoxified hydrolysates (Table 5). In both strains, the most abundant fatty acids were 16:0, 16:1 and 18:1. Detoxification led to an increase in saturated fatty acids, particularly 16:0 and 18:0 and 10:0. The fatty acid 10:0 was detected only in detoxified hydrolysate cultivations. For UFV-1, the saturated fraction increased from 17.3% to 47.1%, whilst in UFV-2, from 21.6% to 38.2%. Overall, the unsaturated fatty acids, mainly 18:1 and 18:2, were reduced in detoxified hydrolysate cultivation. A mixed peak corresponding to 18:2/18:0 was also detected, with relative abundances ranging from 9,74% to 25.5%, depending on the condition and strain. Although this fraction was not included in the calculation of total saturated and unsaturated fatty acids due to its unresolved nature, it represented a considerable portion of the profile. Hence, the results with regard to saturated and unsaturated sums should be considered carefully. Despite these changes, unsaturated fatty acids remained the major fraction in all conditions, particularly, 18:1, which was the most abundant fatty acid across all profiles.

**Table 5:** Fatty acid profile of *C. maltosa* UFV-1 and UFV-2 cultivated in non-detoxified and detoxified malt bagasse lignocellulosic hydrolysates during DCCR model validation. The unresolved 18:2/18:0 fraction was not included in the calculation of total saturated and unsaturated fatty acids

Fatty acid	Fatty acid profile [% (w/w)]			
	*Candida maltosa* UFV-1		*Candida maltosa* UFV-2	
	Non-detoxified	Detoxified	Non-detoxified	Detoxified
10:0	-	0.29 ± 0.11	-	0.40 ± 0.10
12:0	1.17 ± 0.60	0.88 ± 0.24	1.31 ± 0.49	1.49 ± 0.28
14:0	1.83 ± 0.35	2.48 ± 0.35	2.92 ± 0.59	3.72 ± 0.25
16:0	12.31 ± 0.83	27.20 ± 0.58	16.00 ± 2.64	25.18 ± 0.74
17:0	0.58 ± 0.19	1.54 ± 0.04	0.61 ± 0.15	0.86 ± 0.06
18:0	1.35 ± 0.14	14.7 ± 0.84	1.36 ± 0.22	6.58 ± 0.31
14:1	0.80 ± 0.05	0.67 ± 0.03	1.32 ± 0.24	0.96 ± 0.14
16:1	22.70 ± 1.64	11.04 ± 1.44	30.50 ± 4.97	18.00 ± 0.60
18:1	35.40 ± 2.17	38.60 ± 1.90	41.50 ± 8.15	31.60 ± 0.89
18:2/18:0	22.60 ± 1.01	-	25.50 ± 4.75	9.74 ± 0.40
Saturated	17.30 ± 0.38	47.10 ± 0.47	21.60 ± 2.16	38.20 ± 0.39
Unsaturated	58.90 ± 0.48	50.30 ± 0.49	59.00 ± 15.7	50.60 ± 0.20

### Batch bioreactor cultivation of *C. maltosa* UFV-1 using detoxified malt bagasse hemicellulosic hydrolysate

The lipid production of *C. maltosa* UFV-1 after 48 h of cultivation in detoxified malt bagasse hemicellulosic hydrolysate under maximized conditions in a benchtop bioreactor is presented in Table 6. The final biomass reached 79.55 ± 2.52 g/L. The lipid content was 17.21 ± 1.26% (w/w), corresponding to a lipid titer of 13.69 ± 1.01 g/L, a yield of 0.26 ± 0.03, and a productivity of 0.29 ± 0.02 g/L·h. After 48 h of cultivation, no ethanol was detected.

**Table 6:** Physiological parameters of *C. maltosa* UFV-1 after 48 h of cultivation in detoxified malt bagasse hemicellulosic hydrolysates under optimized conditions in a benchtop bioreactor. Values ​​represent mean ± standard deviation

Strain	Final biomass (g/L)	Sugar consumed (g/L)	Lipid content (% m/m)	Lipid titer (g/L)	Lipid Yield ${(Y}_{p/s})$	Lipid Productivity (g/L h)
UFV-1	79.55 ± 2.52	52.18 ± 5.50	17.21 ± 1.26	13.69 ± 1.01	0.26 ± 0.03	0.29 ± 0.02

## Discussion

Production of yeast oil from lignocellulosic biomass hydrolysates is pivotal to meet the rising demand for sustainable oleochemicals. To make microbial lipid-based lignocellulosic biorefinery feasible, the appropriate combination of substrate, product, and organism is mandatory (Nurwono et al. 2023). In this sense, the bioconversion of lignocellulosic byproducts into yeast oil requires robust strains capable of metabolizing pentoses, the main constituents of the hemicellulosic fraction, and tolerating inhibitory compounds generated during pretreatment of lignocellulosic biomasses. In the present study, we isolated yeast strains from soil under sugarcane cultivation, an environment characterized by the recurrent deposition of organic matter, which were able to assimilate and metabolize xylose as a carbon and energy source (Kour et al. 2019). The selected yeasts, *C. maltosa* UFV-1 and UFV-2, displayed tolerance to acetic acid, formic acid, furfural and HMF, beyond achieving high lipid contents from culture medium containing xylose as carbon source. *C. maltosa* has frequently been found in managed soils, representing up to 45.8% of the yeast population in certain agricultural areas (Sláviková & Vadkertiová, 2003). This predominance may be linked to its metabolic versatility, evidenced by its ability to withstand inhibitory compounds and efficiently utilize a wide range of carbon sources, including glucose, fructose, xylose, maltose, sucrose, trehalose, and galactose, as well as hydrocarbons, fatty acids, and aromatic substrates such as phenol and phenylalkanes. Such metabolic versatility allows it to colonize diverse ecological niches and underpins its broad biotechnological applications, including bioremediation, enzymatic bioprospecting, and fermentation processes using industrial byproducts (Chávez-Tinoco et al. 2024; Mauersberger et al. 1996).

The selected yeasts, *C. maltosa* UFV-1 and UFV-2, were able to grow in a medium containing 2 g/L of acetic acid using xylose as the main carbon source maintaining the oleaginous phenotype [≥ 20% (w/w)] even in the presence of 1.0 g/L of acetic acid. These results underscore the potential of both strains, as this acid is the main inhibitor compound in hemicellulosic hydrolysates, remaining in high concentrations even after detoxification steps. It is noteworthy that in pH lower than its pKa (4.76) it can cross the yeast plasma membrane in its undissociated form and dissociate in the cytosol, causing intracellular acidification. This impairs pH homeostasis, in turn yeast growth (Ribeiro et al. 2021). Further studies focusing on understanding the metabolic responses to this acid will bring insights into the mechanisms involved. In oleaginous yeasts, such as *Trichosporon fermentans*, tolerance is associated with active intracellular pH regulation via ATPase pumps and with the redirection of acetyl-CoA toward lipid biosynthesis, which helps relieve metabolic stress and maintain redox balance (Liu et al. 2015; Verduyn et al. 1989). Importantly, *C. maltosa* UFV-1 e UFV-2 also demonstrated the ability to grow in the presence of furfural and HMF, maintaining growth at concentrations of up to 2.0 and 4.0 g/L, respectively, in YNB and YP media. In *Yarrowia lipolytica* and *C. parapsilosis*, the tolerance to these compounds is related to detoxification through NADPH-dependent reductase enzymes (Konzock et al. 2021; Thontowi et al. 2020; Ujor and Okonkwo 2022). Formic acid exhibited higher toxicity, inhibiting growth from 0.5 g/L, possibly due to its lower pKa value, which favors cytosolic acidification (Konzock et al. 2021). As formic acid concentrations increased, growth in YP was slightly greater than in YNB. ​​This difference may be related to the higher buffering capacity of YP medium, which is important for maintaining intracellular pH and redox balance (Hahn-Hägerdal et al. 2005). These results, combined with the ability of *C. maltosa* UFV-1 and UFV-2 to grow and accumulate lipids even in the presence of acetic acid, underscore their potential as promising microbial factories for biorefinery applications based on lignocellulosic biomass conversion.

Adjusting the C:N ratio is a common strategy used to optimize the lipid production in oleaginous yeasts, as nitrogen limitation combined with carbon excess, rewires metabolism toward fatty acid biosynthesis. Under this condition, the inhibition of protein synthesis leads to the accumulation of intermediates such as citrate, which can be converted into acetyl-CoA via ATP-citrate lyase (ACL) (Adrio 2017; Chattopadhyay and Maiti 2021). The results observed for *C. maltosa* UFV-1 showed that lipid content, contrary to biomass and lipid titer, remained stable across different C:N ratios, suggesting that the lipid metabolism regulation appears to be unresponsive to nitrogen availability. Otherwise, *C. maltosa* UFV-2 showed minor variations in lipid content under different C:N ratios, suggesting also a limited influence of nitrogen availability on its lipogenesis. These results indicate that in contrast to other oleaginous yeasts such as *Y. lipolytica and Lipomyces starkeyi*, in which the oleaginous phenotype is well documented, the lipogenesis regulation in *C. maltosa* seems to be different. It is noteworthy that in *Y. lipolytica and L. starkeyi*, the isocitrate dehydrogenase, which is AMP dependent, has its activity decreased in response to nitrogen-limiting conditions, leading to citrate accumulation, which is the the main source of acetyl-CoA for lipid accumulation (Chattopadhyay and Maiti 2021).

To further explore the biotechnological potential of *C. maltosa* UFV-1 and UFV-2, cultivation parameters were evaluated using malt bagasse hemicellulosic hydrolysate as the culture medium. The use of lignocellulosic hydrolysates as fermentation medium is particularly relevant from a process perspective, as it represents an attractive strategy to reduce the costs associated with microbial lipid production. Techno-economic analyses indicate that microbial oil production can be integrated into existing sugarcane biorefineries, where hemicellulosic sugars derived from bagasse are converted into lipids that can subsequently be processed into oleochemicals. Such integration may enhance overall process profitability by sharing infrastructure, utilities, and process streams within the biorefinery (Longati et al. 2022). Both strains exhibited satisfactory growth at pH 6.0 and 7.0, while growth was not observed at pH 2.0 and 3.0. At pH 4.0, only *C. maltosa* UFV-1 was able to grow, and exclusively in the presence of peptone, suggesting that the composition of the nitrogen source influences the adaptation to acid stress conditions. Since the pH range of 6.0 to 7.0 is favorable for *C. maltosa* growth in hemicellulosic hydrolysate, a CCRD was conducted to evaluate the combined effects of initial pH and the P:YE ratio on the performance of both strains. For *C. maltosa* UFV-1, statistically significant models were obtained for both lipid content and titer, while for UFV-2 only lipid content showed a statistically significant response. Consistent with our results, the pH and nitrogen source are recognized as key elements in the regulation of lipogenesis in other oleaginous yeasts, influencing the availability of cytosolic acetyl-CoA and the cellular redox balance by modulating central carbon metabolism, including the activity of alternative acetyl-CoA-generating pathways such as acetyl-CoA synthetase (ACS) and the pyruvate dehydrogenase (PDH) bypass, as well as NADPH-producing reactions like those catalyzed by malic enzyme (Beopoulos et al. 2009; López et al. 2022).

The validation of the models using non-detoxified hydrolysate resulted in values lower than those predicted, possibly due to the complexity of the medium, which includes inhibitory compounds and composition variability (Wang et al. 2024). Although the models were adjusted under controlled conditions, they do not account for factors such as the presence of inhibitors, which can compromise cell viability, and affect the availability of essential cofactors for lipogenesis, such as NADPH (Lyu et al. 2025). Another limiting factor is likely the presence of suspended solids not retained by filtration in the non-detoxified hydrolysate, which can hinder nutrient diffusion or cause physical stress on the cells (Moreno et al. 2022). Detoxification aims to remove or neutralize inhibitory compounds, which contributes to reducing cellular stress and maintaining the physiological integrity of microorganisms (Ujor and Okonkwo 2022). The use of detoxified hydrolysate as substrate led to improvements in all evaluated parameters (biomass, lipid content, titer, productivity, and lipid yield), mainly for *C. maltosa* UFV-1, reaching lipid content and lipid titer of 18.2% and 3.14 g/L, respectively. When cultivated under maximized conditions in a benchtop bioreactor, UFV-1 exhibited increases of 367.9% in biomass and 480% in lipid productivity (79.55 ± 2.52 g/L and 0.29 ± 0.02 g L⁻^1^ h⁻^1^, respectively), highlighting the beneficial effect of improved aeration in the bioreactor system. It is noteworthy that the values of biomass and lipid productivity recorded by *C. maltosa* UFV-1 were superior to those achieved by *Y. lipolytica* W29, cultivated in a stirred-tank bioreactor using eucalyptus bark hydrolysate (biomass titer of 21 g/L and an estimated lipid productivity of 0.06 g L⁻^1^ h⁻^1^ within 48 h) (Dias et al. 2024). Likewise, those values were also superior to the reached by *R. toruloides* cultivated in batch mode using lignocellulosic hydrolysate (approximately 30 g/L of biomass and 15 g/L of lipids after 48 h, corresponding to an estimated lipid productivity of 0.16 g L⁻^1^ h⁻^1^) (Fei et al. 2016).

Glucose was completely consumed in all experiments, confirming the preference of *C. maltosa* for this hexose (Lin et al. 2010). Xylose assimilation was more efficient in the detoxified hydrolysate, especially by *C. maltosa* UFV-1. Ethanol production was observed in Erlenmeyer flask cultivations, likely due to the limited oxygen availability, which favored the fermentative pathway. In contrast, no detectable ethanol was found in the bioreactor cultures, which is consistent with the suitable aeration control in those cultures (Zhou et al. 2023). The fatty acid profile obtained from the cultivation of *C. maltosa* UFV-1 and UFV-2 in detoxified and non-detoxified hemicellulosic hydrolysates was mainly composed of oleic acid (C18:1), palmitic acid (C16:0), palmitoleic acid (C16:1), and stearic acid (C18:0). This profile is characteristic of oleaginous yeasts such as *Y. lipolytica*, *R. toruloides*, and *L. starkeyi* (Poontawee et al. 2023). Such a composition is also relevant from an application perspective. Oils rich in oleic acid (C18:1) present enhanced oxidative stability due to the presence of a single double bond, which is advantageous for cosmetic formulations and improves storage stability in food oils (Ma et al. 2023; Wang et al. 2024). In addition, microbial oils dominated by monounsaturated fatty acids combined with moderate levels of saturated fatty acids (e.g., C16:0 and C18:0) exhibit suitable fuel properties, including favorable cetane numbers and improved oxidative stability for biodiesel production (Falk and Meyer-Pittroff 2004). In addition, a mixed peak corresponding to C18:2 and C18:0 was identified, suggesting a partial co-elution of linoleic and stearic acids. The detoxification promoted an increase in saturated fatty acids, especially C16:0 and C18:0, and led to the detection of capric acid (C10:0), a medium-chain fatty acid absent under non-detoxified conditions. The C18:2/C18:0 fraction represented a substantial portion of the lipid profile, and although it was not possible to distinguish the individual components, its presence suggests a combination of the unsaturated linoleic acid (C18:2), which contributes to membrane fluidity, and the saturated stearic acid (C18:0), which enhances rigidity. The relative abundance of this fraction varied between media, indicating a possible adaptive adjustment. In the detoxified medium, the higher proportion of C18:0 may have enriched the saturated portion of this fraction, promoting greater membrane stability under less stressful conditions. In contrast, in the non-detoxified medium, the need to maintain fluidity in response to inhibitors may have favored a higher proportion of C18:2. This modulation of the ratio between unsaturated and saturated fatty acids is recognized as part of a membrane remodeling mechanism in yeasts, which is essential to counteract chemical and physical stressors such as those present in lignocellulosic hydrolysates (Ballweg et al. 2020).

## Conclusions

In this study, a targeted bioprospecting approach was employed to isolate and screen yeasts from soil samples, combining morphological grouping, PCR-fingerprint, and functional assays for xylose assimilation, growth in the presence of hydrolysate-derived compounds, and lipid accumulation. We demonstrated that *C. maltosa* UFV-1 and UFV-2 are promising strains for application in bioprocesses based on the conversion of hemicellulosic hydrolysate sugars into lipids, ethanol and biomass under oxygen-limiting conditions. Both strains were able to grow on xylose and acetic acid and maintained lipid accumulation under these cultivation conditions. Notably, UFV-1 achieved a highest biomass titer, enhanced growth in detoxified hydrolysate, and significant lipid productivity in bioreactor cultivations conducted under aerobic conditions. The fatty acid profile, predominantly composed of C18:1, C16:0, C18:2, and C18:0, is compatible with applications in the food, cosmetic, oleochemical, and biofuel sectors. Future studies should focus on bench-scale bioreactor cultivations with refined control of culture parameters, as well as on metabolic and genetic engineering strategies to redirect intracellular fluxes toward targeted products. Leveraging the intrinsic robustness, high biomass production, and versatile metabolism of these strains could maximize yields of desired bioproducts and expand their biotechnological applicability. From an industrial perspective, the production of microbial oil by *C. maltosa* UFV-1 using detoxified malt bagasse hydrolysate could be integrated into brewery operations through the implementation of an adjacent biorefinery unit designed to valorize malt bagasse via acid hydrolysis. In this configuration, pretreatment, hydrolysis, detoxification, and fermentation steps could be integrated with existing industrial utilities, followed by cell separation and oil recovery processes for lipid extraction. Such an approach could contribute to the circular use of brewery by-products while reducing logistical costs associated with residue management.

## Supplementary Information

Below is the link to the electronic supplementary material.

Supplementary file1 (PDF 41 KB)

Supplementary file2 (PDF 40 KB)

Supplementary file3 (PDF 124 KB)

## Data Availability

Data generated during this study are available from the corresponding author on reasonable request.
