# Supplementary figures and images for: Isolation of Candida maltosa strains able to achieve a high lipid productivity from malt bagasse hemicellulosic hydrolysate
Source: World J Microbiol Biotechnol. 2026 Mar 30;42(4):167. doi: 10.1007/s11274-026-04919-9 (PMC13035538; doi:10.1007/s11274-026-04919-9)

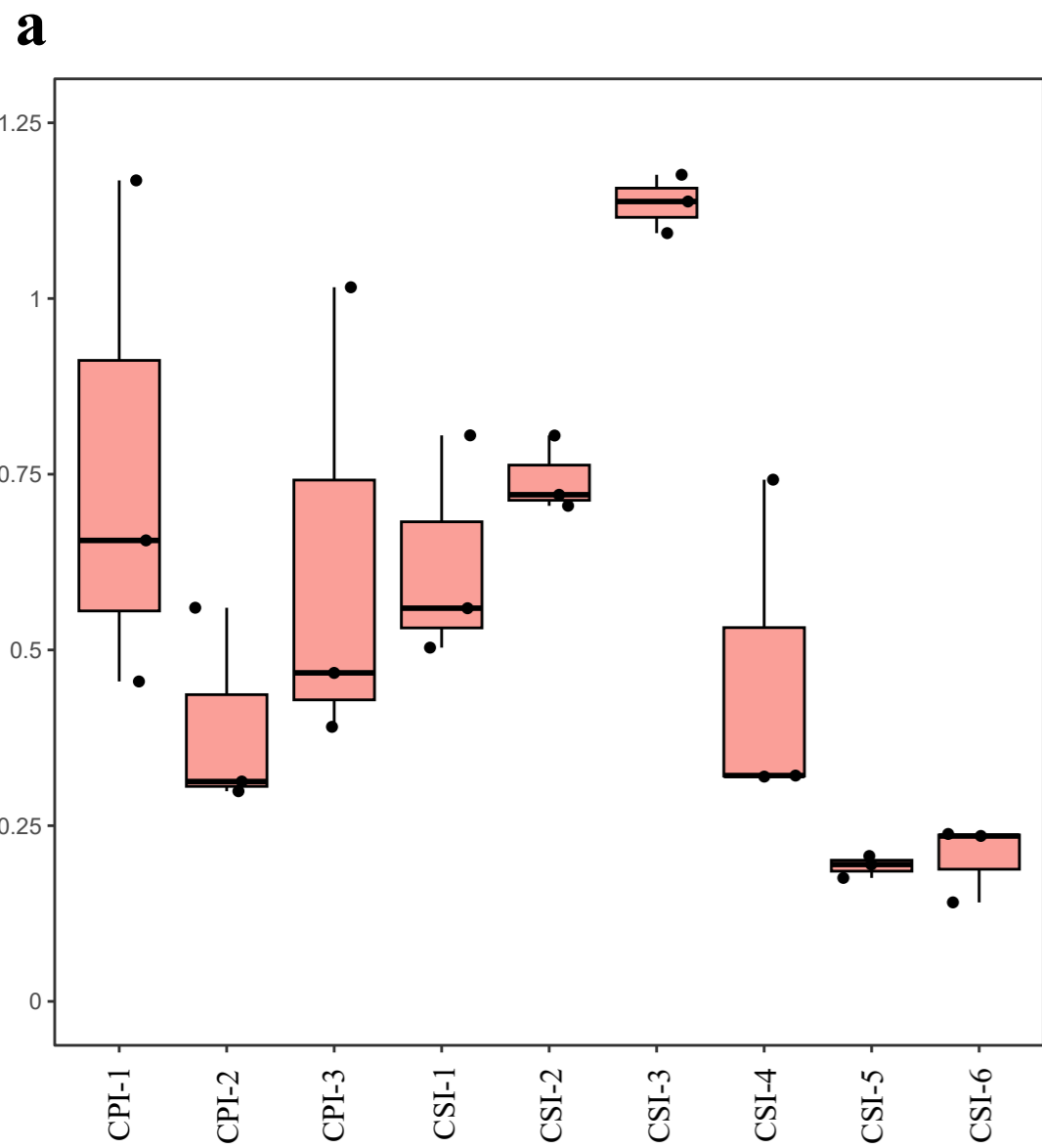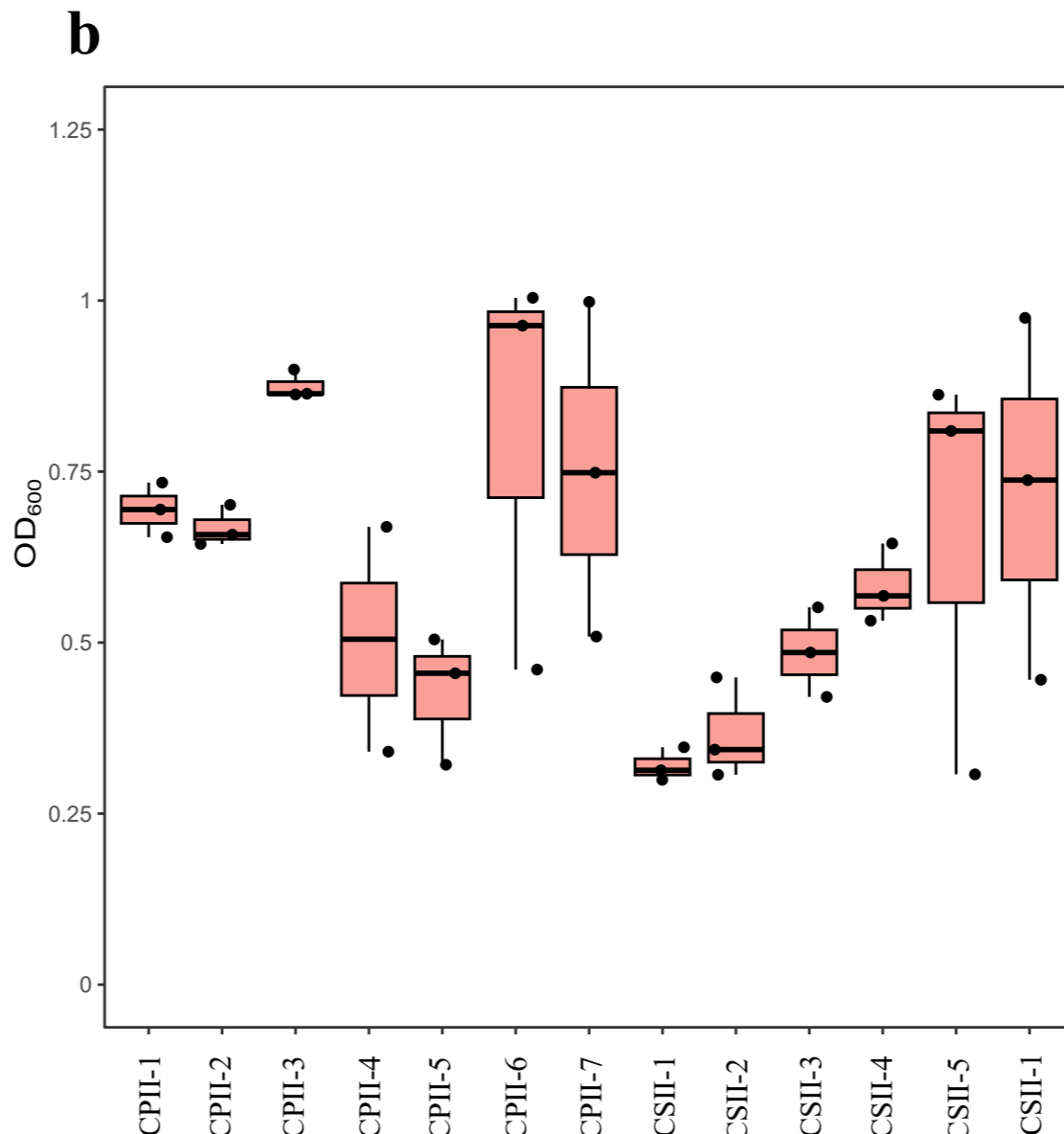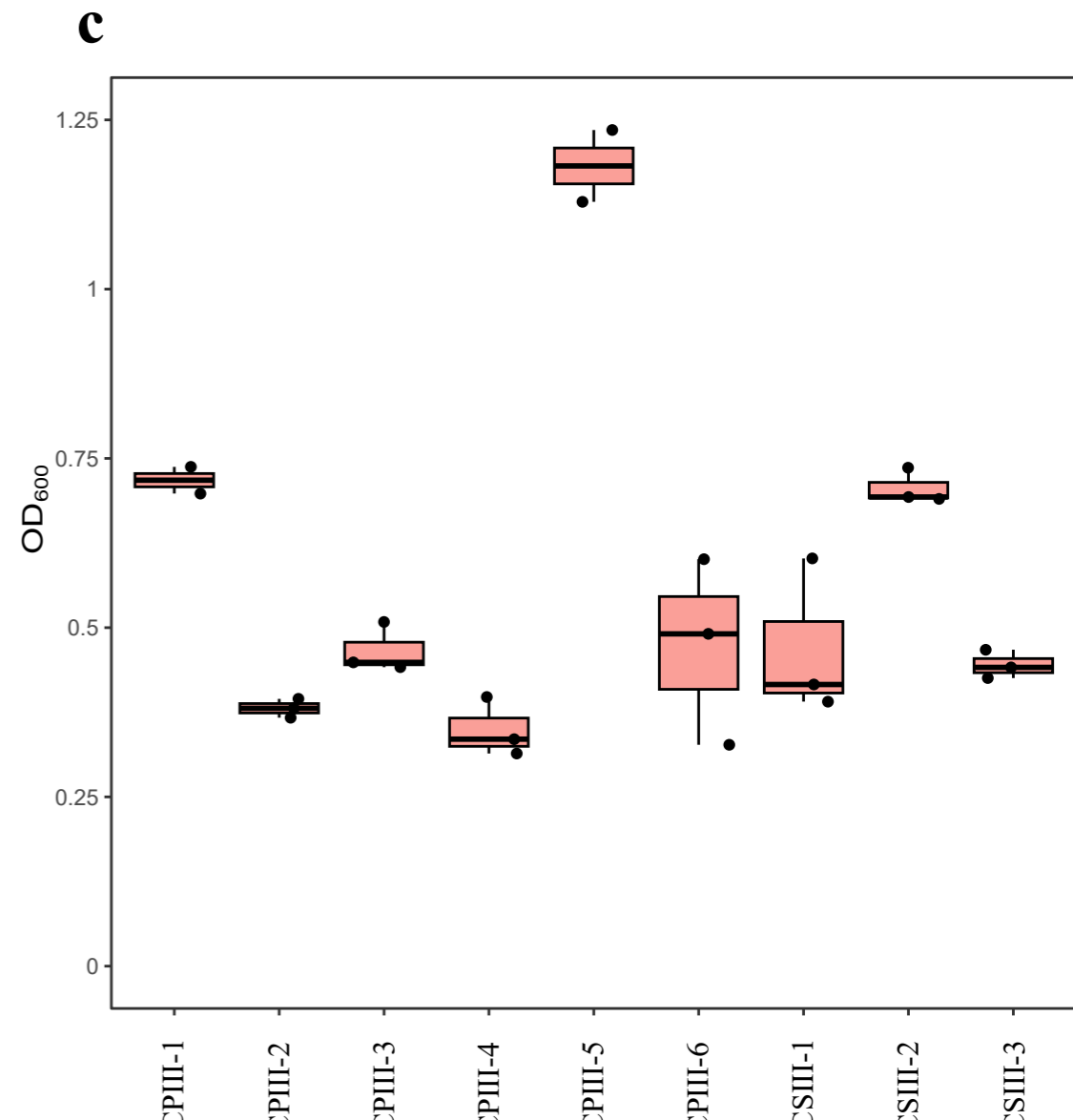

Supplement: Supplementary file 1 — Supplementary file1 (PDF 41 KB) [file 11274_2026_4919_MOESM1_ESM.pdf]

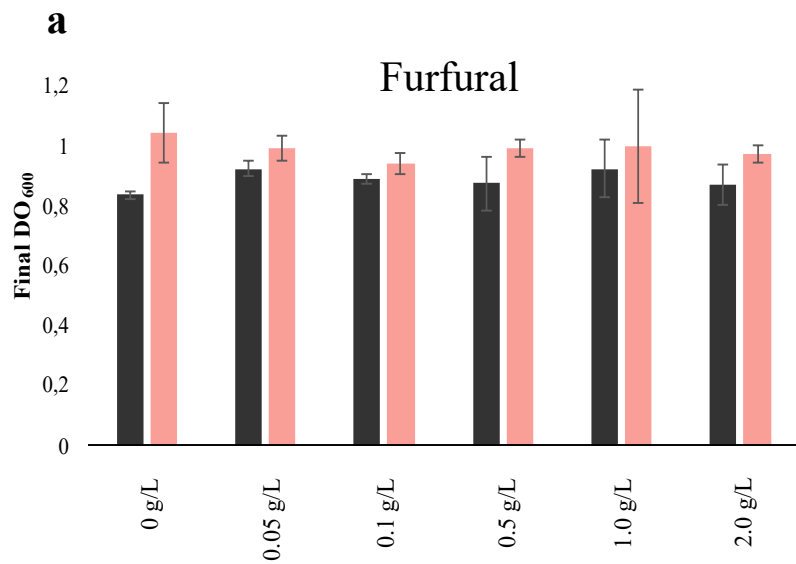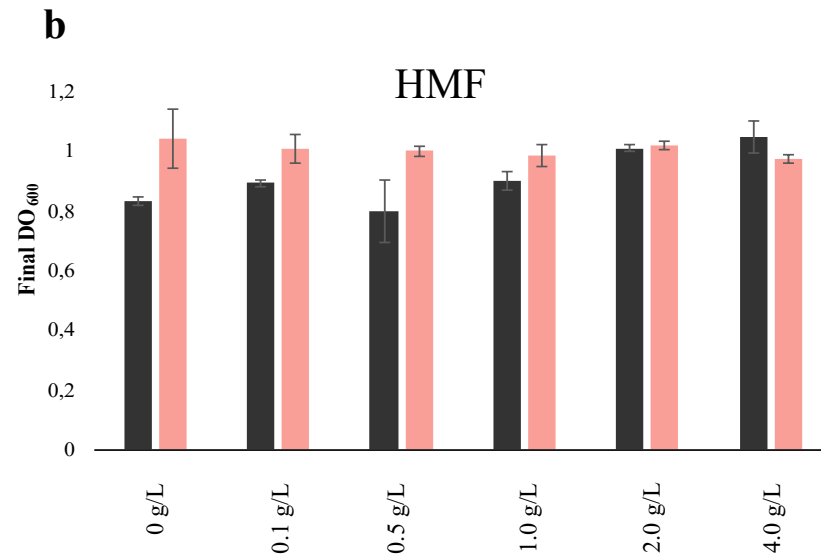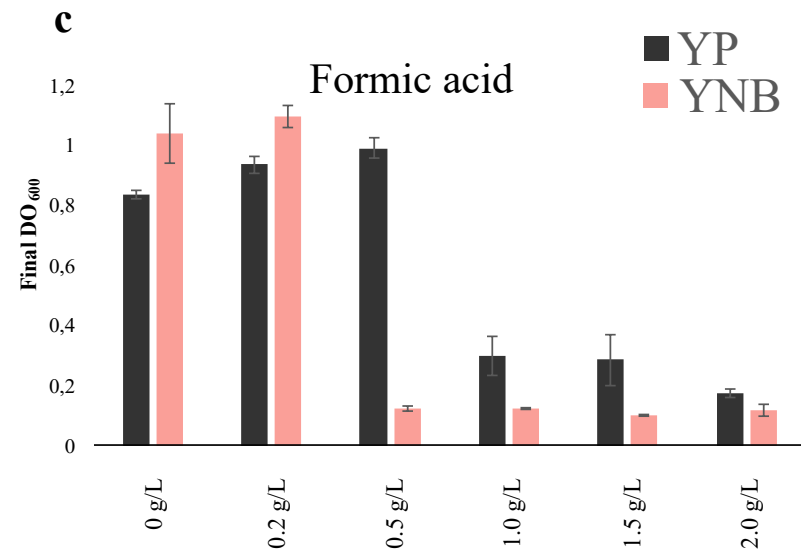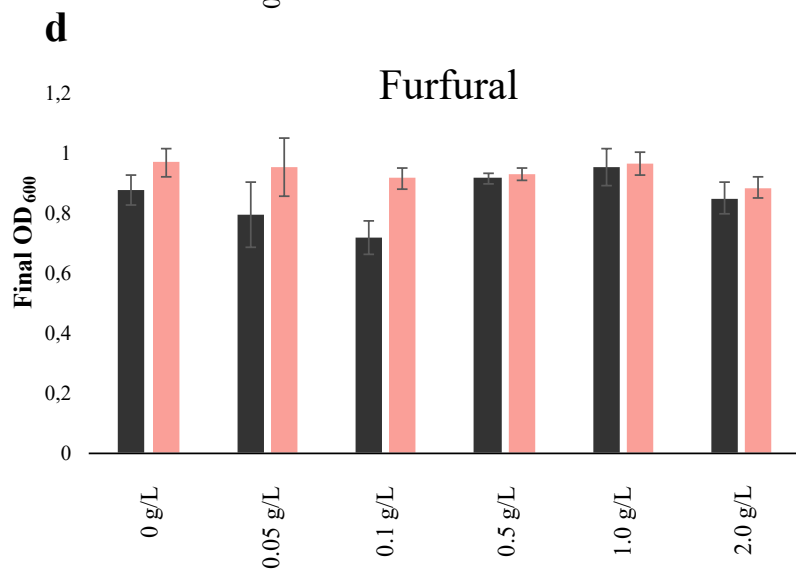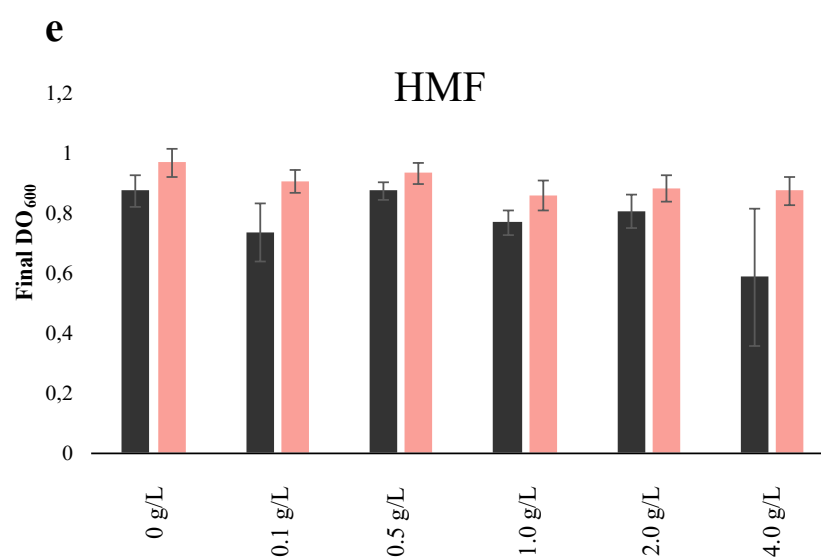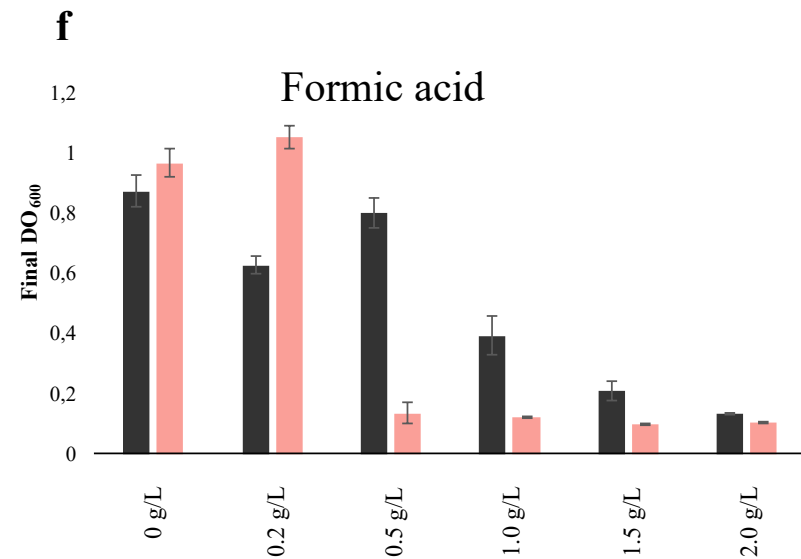

Supplement: Supplementary file 2 — Supplementary file2 (PDF 40 KB) [file 11274_2026_4919_MOESM2_ESM.pdf]

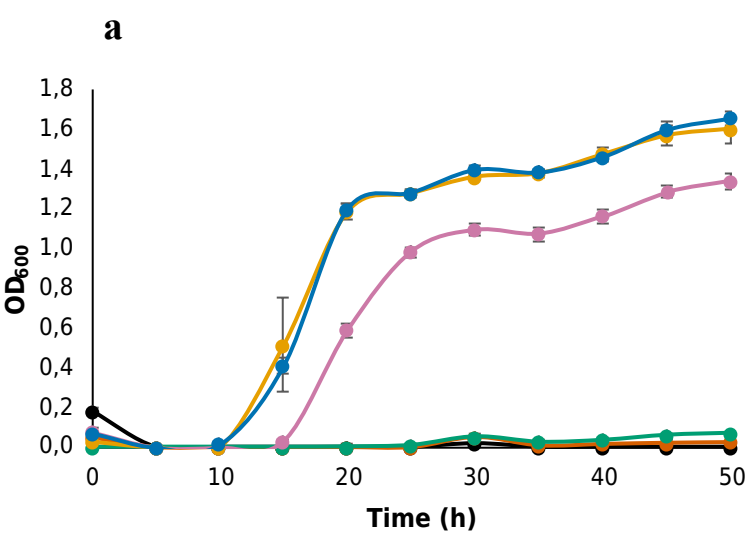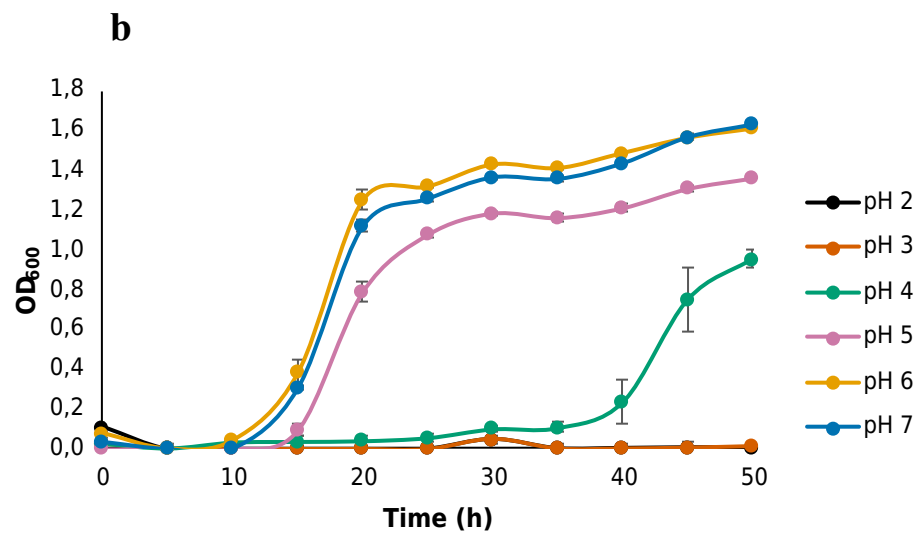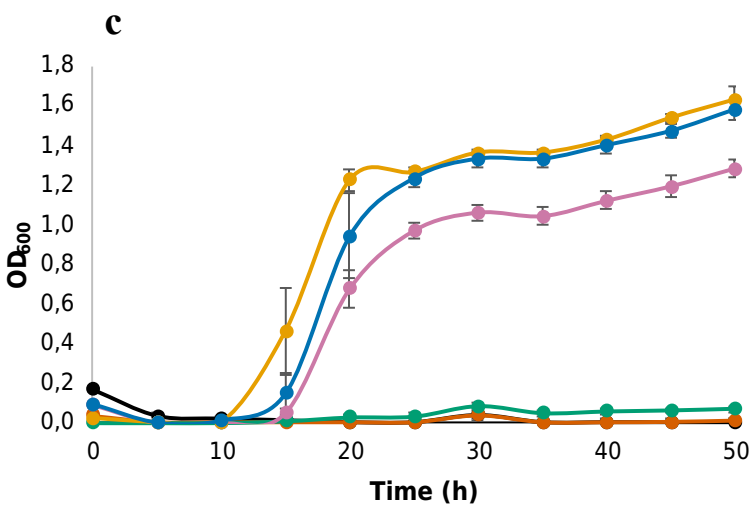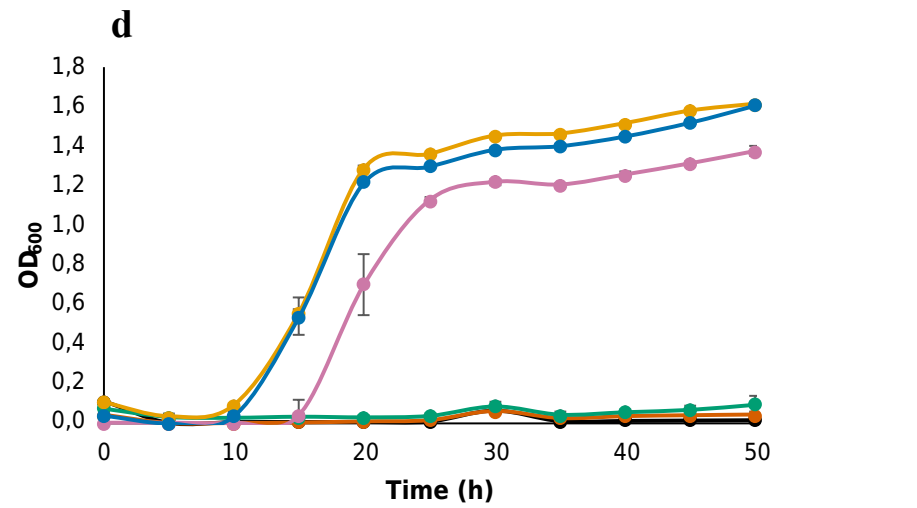

Supplement: Supplementary file 3 — Supplementary file3 (PDF 124 KB) [file 11274_2026_4919_MOESM3_ESM.pdf]
